# CellVGAE: an unsupervised scRNA-seq analysis workflow with graph attention networks

**DOI:** 10.1093/bioinformatics/btab804

**Published:** 2021-12-02

**Authors:** David Buterez, Ioana Bica, Ifrah Tariq, Helena Andrés-Terré, Pietro Liò

**Affiliations:** Department of Computer Science and Technology, University of Cambridge, Cambridge CB3 0FD, UK; Department of Engineering Science, University of Oxford, Oxford OX1 3PJ, UK; The Alan Turing Institute, London NW1 2DB, UK; Computational and Systems Biology Program, Department of Biological Engineering, Massachusetts Institute of Technology, Cambridge, MA 02142, USA; Department of Computer Science and Technology, University of Cambridge, Cambridge CB3 0FD, UK; Department of Computer Science and Technology, University of Cambridge, Cambridge CB3 0FD, UK

## Abstract

**Motivation:**

Single-cell RNA sequencing allows high-resolution views of individual cells for libraries of up to millions of samples, thus motivating the use of deep learning for analysis. In this study, we introduce the use of graph neural networks for the unsupervised exploration of scRNA-seq data by developing a variational graph autoencoder architecture with graph attention layers that operates directly on the connectivity between cells, focusing on dimensionality reduction and clustering. With the help of several case studies, we show that our model, named CellVGAE, can be effectively used for exploratory analysis even on challenging datasets, by extracting meaningful features from the data and providing the means to visualize and interpret different aspects of the model.

**Results:**

We show that CellVGAE is more interpretable than existing scRNA-seq variational architectures by analysing the graph attention coefficients. By drawing parallels with other scRNA-seq studies on interpretability, we assess the validity of the relationships modelled by attention, and furthermore, we show that CellVGAE can intrinsically capture information such as pseudotime and NF-ĸB activation dynamics, the latter being a property that is not generally shared by existing neural alternatives. We then evaluate the dimensionality reduction and clustering performance on 9 difficult and well-annotated datasets by comparing with three leading neural and non-neural techniques, concluding that CellVGAE outperforms competing methods. Finally, we report a decrease in training times of up to × 20 on a dataset of 1.3 million cells compared to existing deep learning architectures.

**Availabilityand implementation:**

The CellVGAE code is available at https://github.com/davidbuterez/CellVGAE.

**Supplementary information:**

Supplementary data are available at *Bioinformatics* online.

## 1 Introduction

scRNA-seq allows gene expression to be quantified at the level of individual cells; however, it introduces new challenges, as technical and biological limitations contribute to noisier and more complex data than previous sequencing techniques (Chen *et al.*, 2019). A recent study found over 19 000 scRNA-seq studies as of October 2020 (Pasquini *et al.*, 2021), and another identified over 1000 scRNA-seq tools as of September 2021 (Zappia and Theis, 2021), reflecting rapid iterative progress in the field. Non-neural tools are the most widespread, most likely for their simplicity and interpretability. However, it is typically beyond the capabilities of algorithms such as t-SNE (van der Maaten and Hinton, 2008) and UMAP (McInnes *et al.*, 2020) to perform interpretable dimensionality reduction from the high-dimensional gene space. Consequently, this task, as well as denoising and imputation are often handled by neural networks, especially Variational Autoencoders (VAEs) such as scVAE (Grønbech *et al.*, 2020), Deep Count Autoencoder (Eraslan *et al.*, 2019), scVI (Svensson *et al.*, 2020), (Lopez *et al.*, 2018) and DiffVAE (Bica *et al.*, 2020). A popular alternative is graph-based clustering, popularized by Seurat (Stuart *et al.*, 2019). Recent developments such as self-assembling manifolds (SAM) (Tarashansky *et al.*, 2019) have since largely superseded Seurat and are competitive with neural networks.

In this work, we investigate a new machine learning approach with applications to dimensionality reduction and clustering. Based on the recent interest in both graph-based scRNA-seq clustering and graph neural networks, we propose a neural model that is built upon the variational graph autoencoder (VGAE) (Kipf and Welling, 2016b) with graph attention layers (GAT) (Veličković *et al.*, 2018), named CellVGAE. Compared to other neural models which learn exclusively from the gene expression values, CellVGAE leverages the connectivity between cells (represented as a graph) as an inductive bias to perform convolutions on a non-Euclidean structure, thus subscribing to the geometric deep learning paradigm. We use k-nearest neighbour (KNN) and Pearson correlation graphs (referred to as PKNN), based on their efficient implementations and widespread use. While methods based on Euclidean distance are popular, Pearson correlation has recently been argued to be preferable for scRNA-seq data (Kim *et al.*, 2019).

There are several reasons motivating the introduction of a graph neural network (GNN) methodology. Firstly, the graph can support learning, acting as a valuable inductive bias and allowing the model to exploit relationships that are impossible or harder to model by the simpler dense layers. Secondly, graphs are generally more interpretable and visualizable; the GAT (Graph Attention Network) framework made important steps in bringing these desirable features to machine learning, a trait not shared by traditional (non-graph) methods. The attention mechanism is argued to (i) improve task performance, (ii) stabilize learning (reduce variance), and (iii) provide an extra layer of interpretability. Thirdly, by jointly using the variational autoencoder and graph neural networks, we allow future studies to exploit advances in both of these active research subjects, such as the newly published GATv2 (Brody *et al.*, 2021) convolutional layer (already integrated into CellVGAE).

For our evaluation of CellVGAE we compare with existing methods that: (i) achieve state-of-the-art performance on dimensionality reduction and clustering (unsupervised learning), (ii) are open source and (iii) are recognized in the scRNA-seq community. We select SAM and DiffVAE as recent advances in their respective fields and for their performance, and scVI for its popularity.

## 2 Materials and methods

In this work, we assume simple graphs (undirected, unweighted, without loops or multiple edges), defined as a tuple G=(V,E), where V is the set of vertices or nodes $\left\{v_{0},v_{1},\ldots \right\}$ and E is the set of edges between nodes, E⊆V×V. A common representation is given by a graph’s adjacency matrix **A** (whose elements *a_ij_* = 1 if $\langle v_{i},v_{j}\rangle \in E$, with *a_ij_* = 0 otherwise). For GNNs, we also assume that *D*-dimensional node features are represented by a *N *×* D* matrix **X**, where N=|V|.

### 2.1 Variational graph autoencoder

The variational graph autoencoder (VGAE) is an unsupervised framework introduced in (Kipf and Welling, 2016b). Like the standard VAE, the VGAE has two components: an encoder and a decoder, which are trained to learn latent variables **z**, aggregated in an *N *×* L* matrix **Z**, where *L* is the number of latent dimensions. The encoder or inference model is similar to the VAE, but does not depend only on the signal **X**, but also on the graph **A**:

(1) $$\begin{array}{cc} q(Z|X,A)=\prod_{i=1}^{N}q(z_{i}|X,A) & q(z_{i}|X,A)=N(z_{i}|\mu_{i},\text{diag}(\sigma_{i}^{2})) \end{array}$$

In the original formulation, the parameters μ,σ are learnt by graph convolutional networks (GCN) (Kipf and Welling, 2016a); formally $\mu ={\text{GCN}}_{\mu }(X,A)$ and $\text{log}\;\sigma ={\text{GCN}}_{\sigma }(X,A)$, where **X** are features learnt by previous convolutional layers.

The decoder, or generative model, simply reconstructs an adjacency matrix using the inner product of latent variables:

(2) $$\begin{array}{cc} p(A|Z)=\prod_{i=1}^{N}\prod_{j=1}^{N}p(A_{ij}\;|\;z_{i},z_{j}) & p(A_{ij}=1\;|\;z_{i},z_{j})=\sigma (z_{i}^{⊤}z_{j}) \end{array}$$

Here, *σ* is the logistic sigmoid function. Also, note that only the graph structure is reconstructed (not the node features). The loss function is of the form:

(3) $$L=\underset{L_{\text{RECON}}}{\underset{︸}{E_{q(Z|X,A)}\left[\text{log}\;p(A|Z)\right]}}-\underset{L_{\text{REG}}}{\underset{︸}{D_{\text{KL}}\left[q(Z|X,A)∥p(Z)\right]}}$$

where the two components are the reconstruction loss $L_{\text{RECON}}$ and a regularization term $L_{\text{REG}}$. In the standard VGAE framework, the regularization is given by the Kullback-Leibler divergence $D_{\text{KL}}$ between q(·) and p(·).

### 2.2 Graph attention networks

Graph attention networks (GAT) are a powerful neural framework for graph-structured data, initially designed for supervised and semi-supervised learning, manifested through the *graph attention layer*, capable of performing self-attention on graph nodes. Given a set of node features $h=\{h_{1},h_{2},\ldots ,h_{N}\}$ with $h_{i}\in R^{D}$, a shared linear transformation parameterized by a weight matrix **W** is applied to all nodes, followed by a learnable attention mechanism *a* applied pairwise to the scaled node features: $e_{ij}=a\left(Wh_{i},Wh_{j}\right)$, where $W\in R^{D^{\prime }\times D}$ and $a:R^{D^{\prime }}\times R^{D^{\prime }}\to R$ assuming D′ is the output dimension of the nodes. The number *e_ij_* can be interpreted as the contribution of node *j’*s features to node *i*. To leverage the graph structure, the computations are limited to the neighbourhood $N_{i}$ of a node *i*. Finally, the outputs are normalized using the softmax function:

(4) $$\alpha_{ij}=\frac{\;\text{exp}\;\left(\text{LeakyReLU}\left(a^{⊤}\left[Wh_{i}∥Wh_{j}\right]\right)\right)}{\sum_{k\in N_{i}}\;\text{exp}\;\left(\text{LeakyReLU}\left(a^{⊤}\left[Wh_{i}∥Wh_{k}\right]\right)\right)}$$

where $\cdot^{⊤}$ is transposition, ∥ is concatenation, LeakyReLU is a variant of ReLU that allows a non-zero gradient when the argument is ≤0 (Maas *et al.*, 2013), and *α* are attention coefficients. The normalized coefficients are used together with a learnable linear combination of the neighbouring features (usually after applying a non-linearity *σ*) to produce the output node features. As an extension, the attention mechanism can be applied independently *K* times (multi-head attention), in two ways:

(5) $$\begin{array}{cc} h\prime_{i}=\overset{K}{\underset{k=1}{\|}}\sigma \left(\sum_{j\in N_{i}}\alpha_{ij}^{k}W^{k}h_{j}\right) & h\prime_{i}=\sigma \left(\frac{1}{K}\sum_{k=1}^{K}\sum_{j\in N_{i}}\alpha_{ij}^{k}W^{k}h_{j}\right) \end{array}$$

The left formulation is appropriate for the hidden (inner) layers as it outputs the concatenated hidden representations of dimension K·D′. The variation on the right uses the mean of all the attention heads and is suitable for the last (output) layer.

### 2.3 CellVGAE architecture

CellVGAE’s encoder is based on graph attention layers. Using the established notation, where $X_{0}$ is the initial set of node features and ${\text{GAT}}_{i}^{\left(K\right)}$ represents the *i*th layer with *K* attention heads, we define the first and subsequent inner neural layers for an architecture with *N* inner layers:

(6) $$X_{i}=\{\begin{array}{cc} {\text{GAT}}_{i}^{\left(K\right)}(X_{0},A) & \text{if}\;i=1\\ {\text{GAT}}_{i}^{\left(K\right)}(\text{ReLU}(X_{i-1}),A) & \text{if}\;1<i\leq N \end{array}$$

The inner layers concatenate the representations learnt by multi-head attention, thus after the first layer, the number of output dimensions is $K\cdot D_{i}$, with *D_i_* the output dimension for each layer. The number of heads *K* can be different for each layer. The final two layers learn the parameters μ,σ and follow the second branch of Equation 6:

(7) $$\begin{array}{cc} \mu ={\text{GAT}}_{\mu }^{\left(K\right)}(\text{ReLU}(X_{N}),A) & \;\text{ log}\;\sigma ={\text{GAT}}_{\sigma }^{\left(K\right)}(\text{ReLU}(X_{N}),A) \end{array}$$

with the exception that they use mean instead of concatenation.

Apart from their demonstrated performance, GAT layers are appealing for several reasons. Since the coefficients indicate the contribution of nodes in a pairwise manner, they can be visualized as a weighted graph between the cells. We confirm that CellVGAE attention models non-spurious and accurate relationships in Supplementary Information SD and SE. Secondly, as GAT layers use a linear transformation under the hood, a traditional layer weights analysis can be carried out, in a manner very similar to DiffVAE (detailed in Section 3). Currently, CellVGAE provides the users with the choice of GCN, GAT and GATv2 layers. A comparative study is provided in Supplementary Information SH.

We use the same inner product decoder as the original VGAE, as well as the standard loss that combines regularization and reconstruction terms:

(8) $$L(X)=L_{\text{REG}}+\underset{L_{\text{RECON}}}{\underset{︸}{E_{z∼q(Z|X,A)}[\text{log}\;p(A|Z)]}}$$

with the mention that $L_{\text{REG}}$ can use any appropriate loss from the literature that minimizes the divergence between the learnt and prior distributions. CellVGAE can be used with the KL (Kingma and Welling, 2014) and MMD (Zhao *et al.*, 2017) losses, normally implemented as:

(9) $$\begin{array}{c} L_{\text{REG}}=-D_{\text{KL}}\left[q(Z|X,A)∥p(Z)\right]\\ \text{or}\\ L_{\text{REG}}=-D_{\text{MMD}}(q(Z)∥p(Z)) \end{array}$$

$L_{\text{RECON}}$ is implemented as a binary cross entropy loss with negative sampling, where ${\hat{y}}^{\text{pos}}$ is the decoder output for positive (real) edges, ${\hat{y}}^{\text{neg}}$ is the decoder output for (randomly sampled) negative edges, and *E* is the total number of edges:

(10) $$\begin{array}{c} L_{\text{RECON}}=-\frac{1}{E}\left(\sum_{i}\;\text{log}\;({\hat{y}}_{i}^{\text{pos}})+\sum_{j}\;\text{log}\;(1-{\hat{y}}_{j}^{\text{neg}})\right) \end{array}$$

Optionally, CellVGAE can use a feature reconstruction neural network $f_{\text{RECON}}$ in the decoder, addressing a limitation of the original VGAE framework, such that the loss becomes:

(11) $$L\prime (X)=L(X)+\text{MSE}(f_{\text{RECON}}(Z),X)$$

where MSE is the mean squared error.

### 2.4 CellVGAE workflow

The key idea is augmenting the gene expression matrix with cell connectivity information in the form of a graph. In this work, the used graphs take the form of KNN graphs based on Euclidean distance and PKNN graphs built using Pearson correlation, although the CellVGAE interface allows other metrics (such as Manhattan distance, cosine similarity, etc.) and completely custom graphs to be provided. In this formulation, the gene expression values associated with a cell are used as node features for the VGAE. For this article, we log-normalized the expression matrix, a widespread practice in the scRNA-seq community (Booeshaghi and Pachter, 2021) and applied min-max scaling, which is also common in machine learning applications. Furthermore, for practical reasons such as training efficiency and balancing the number of features compared to the number of cells we generally select a number of top highly variable genes (HVGs) to use as node features, a standard practice for both neural and non-neural tools (Yip *et al.*, 2019). The graph can be built from the original high-dimensional data, projections of the data to a lower number of dimensions (for example using Principal Component Analysis—PCA), or from a number of HVGs that can be different from the number of HVGs used as node features. Since we frequently use this last hyperparameter we refer to it as KHVG, the number of HVGs used to build the graph. As an unsupervised method, CellVGAE training is performed on the whole dataset. For evaluation purposes, a held-out test set is first used for each dataset to determine convergence and prevent overfitting, as well as suggest optimal graph settings (i.e. k and KHVG).

**Fig. 1.**
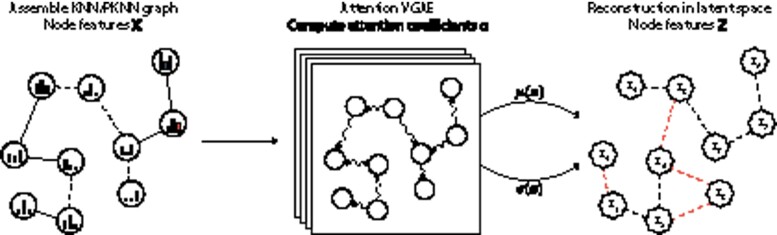
High-level overview of a typical CellVGAE workflow. The input consists of a KNN or PKNN graph derived from the transcriptomics data, where the associated node features **X** are the gene expression values. Training the VGAE (shown with multiple layers) follows the computational steps outlined in ‘*CellVGAE architecture*’, outputting the latent node features **Z** and reconstructing the original graph by edge prediction. Dimensionality reduction is achieved by selecting a number of latent dimensions that is smaller than the original input dimension. The resulting latent node features can be used for downstream analysis

By propagating the transcriptomics information according to the cell connections, CellVGAE learns to reconstruct the original graph from the lower-dimensional latent space (Supplementary Information SM), producing high-quality compressed representations that can be used for downstream analysis, detailed in Section 3. The process is schematically represented in Figure  1. 

## 3 Results

### 3.1 Accurate identification of clusters on challenging datasets

One of the motivating factors behind the SAM algorithm is the inability of the existing methods to analyse a novel scRNA-seq dataset of *Schistosoma mansoni* (including cell types such as ϵ,δ′ and *μ*). More specifically, Tarashansky *et al.* (2019) showed that commonly used methods like PCA, Seurat and SIMLR (Wang *et al.*, 2017) fail to distinguish any cluster formations. In turn, this renders tasks like cell (sub)type identification and finding marker genes difficult using current tools. To formalize this notion of difficulty, the authors introduce an unsupervised metric called *network sensitivity*, which measures the changes in cell-to-cell distances on randomly selected subsets of the gene expression matrix. An intuitive explanation of a high sensitivity score is that changes in the used features lead to a significantly different topological network. On the other hand, datasets robust to the said feature selection have many genes (signals) reinforcing the same structure, thus resulting in a low sensitivity score. The exact sensitivity ranking and numerical values for the *S.mansoni*, *Macrophages* and the other 9 datasets used in *Results* are available in (Tarashansky *et al.*, 2019) and reproduced in Supplementary Figure S1. The *S.mansoni* dataset tops the network sensitivity chart by a significant lead, followed by the *Macrophages* dataset, which is in turn about twice as difficult as the next most challenging dataset.

#### 3.1.1 The *S.mansoni* dataset

**Fig. 2.**
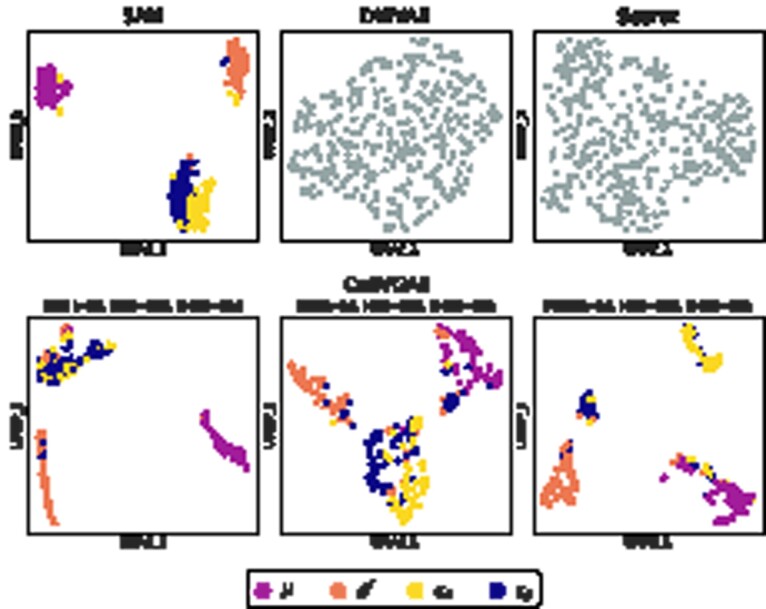
UMAP plot of the cell embeddings for the four evaluated methods on ‘*S.mansoni*’, CellVGAE being illustrated with three different configurations that capture alternative views of the data. DiffVAE and Seurat representations do not distinguish any clusters and make the application of density-based clustering impossible. For CellVGAE, the first panel to the left depicts three clearly delimited clusters, just as SAM with density-based clustering, and moreover, we observe 95.30% overlap for the *ϵ* cluster (142 common cells out of 149), 94.62% overlap for the *μ* cluster (88 common cells out of 93) and 76.04% overlap for the δ′ cluster (73 common cells out of 96), where the overlap per cent is calculated as the number of common cells over the number of cells in the original SAM cluster. To summarize, this leads to an average of 88.65% overlap between the clusters, with similar percentages for the other configurations. In this first CellVGAE configuration, the $\epsilon_{\alpha }$ cells tend to be present at the bottom of the larger *ϵ* cluster; however, the separation of the two subpopulations can be seen in the second panel (centre), where again three clusters are found by HDBSCAN and the two subpopulations are distinguished in the overlay. On a different note, the shape of the clusters in the first panel is more linear, while for the other two configurations the shapes are irregular. Finally, we observe that with different parameters (last panel, right) CellVGAE is capable of largely separating all four clusters, being the only method that we are aware of with this capability. In this configuration, the $\epsilon_{\beta }$ cells are the most difficult to cluster, since they are highly related to $\epsilon_{\alpha }$ cells, as can be seen in the corresponding (yellow) cluster. The few cells that do not agree with SAM are generally towards the margin of their clusters, indicating transcriptomic similarity to the cluster they belong to as well as to neighbouring clusters

Figure  2 presents the UMAP plots of the evaluated dimensionality reduction techniques including multiple configurations of CellVGAE, with a colour overlay provided by Louvain clustering as computed by SAM. The methods are run with the recommended hyperparameters, set to be equivalent between models when possible. The CellVGAE clusters are determined with HDBSCAN (McInnes *et al.*, 2017), an unsupervised density-based clustering algorithm. We emphasize that the SAM clustering is derived purely computationally and should not necessarily be equated to the ground truth for this dataset.

SAM, the current gold standard for this dataset, finds three clearly separated clusters (*μ*, δ′ and *ϵ* cells) that can be easily identified by density-based clustering. Louvain clustering further clarifies two subpopulations of *ϵ* cells: $\epsilon_{\alpha }$ and $\epsilon_{\beta }$; however, these are not precisely separated. It is clear that the DiffVAE and Seurat representations do not distinguish any clusters and make the application of density-based clustering impossible. In contrast, the three illustrated CellVGAE settings manage to discover the underlying clustering, highlighting different aspects of the data. These are illustrated and discussed in Figure  2.

#### 3.1.2 The *Macrophages* dataset

For the second case study, we analyse the *Macrophages* dataset, where the authors examined the NF-ĸB activation dynamics and transcriptional profile of 823 macrophages treated with lipopolysaccharide (LPS) (Lane *et al.*, 2017). We follow the methodology of Tarashansky *et al.* to remove cell cycle effects from 637 cells which were imaged for NF-ĸB response after 75, 150 and 300 min. The resulting expression matrix is used as input to CellVGAE and the other evaluated neural algorithms. Tarashansky *et al.* further argue that the cells can be grouped in two clusters by their activated signalling pathways: Myd88 and TRIF (MT) and only Myd88 (M).

**Fig. 3.**
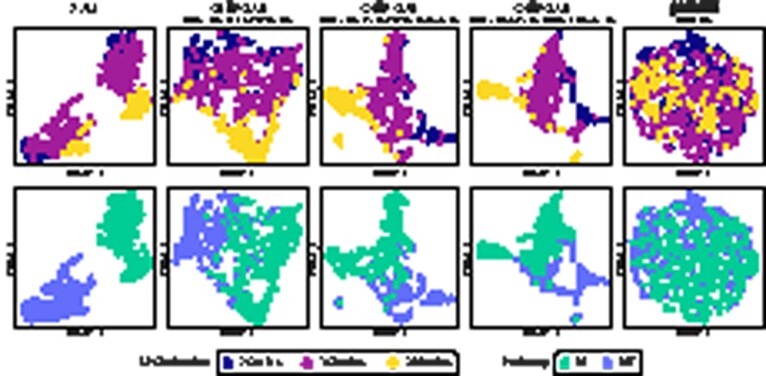
UMAP plots of the ‘*Macrophages*’ dataset for SAM, CellVGAE and DiffVAE. SAM was previously the only known algorithm capable of ordering the cell embeddings by the elapsed time since LPS induction (measured at three time points). scVI is not evaluated as it is only applicable to the raw counts, which are not provided for this dataset. DiffVAE does not distinguish any LPS induction or NF-ĸB activation patterns when applied to an expression matrix of 2500 HVGs (the same outcome is achieved for models with HVGs ranging from 100 to 5000, Supplementary Fig. S2)

As seen in Figure  3, the three illustrated CellVGAE models prefer to first and foremost cluster based on the NF-ĸB activation dynamics, such that cells belonging to the same chronological grouping are generally in close proximity. At the same time, the CellVGAE representation respects the two pathway clusters found by SAM. The set of cells imaged after 300 min can be almost completely separated (fourth column in Fig.  3), although a small cluster of ‘300 mins’ cells is separated from the main agglomeration as it is part of the different MT cluster. However, when using less transcriptomics information but otherwise identical settings (third column), or similarly for the settings of the second column, there is a continuum between the clusters emphasizing the preference to group by time since LPS induction. Thus, the continuous, non-interrupted topology is required to characterize the transcriptional profile and activation dynamics simultaneously.

#### 3.1.3 The *PBMC3k* dataset

**Fig. 4.**
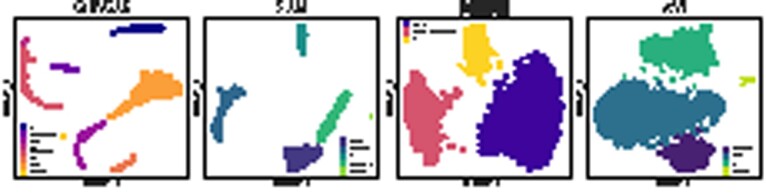
Clustering performance of CellVGAE, SAM, DiffVAE and scVI on the ‘*PBMC3k*’ dataset (UMAP). Labels are determined by the highest overlap with Seurat clusters. SAM cannot distinguish the NK population from the CD8 T population, both being relatively large, and neither the more finely grained Monocytes and DC cell populations. Similarly, DiffVAE groups NK, CD4 T and CD8 T cells together in a single cluster, whereas Monocytes and DC are recognized as a single cluster like in SAM, the purest cluster being the third (B cells). The clustering of scVI is similar to DiffVAE, as expected, but with a clearly separated Platelets cluster. Numerically, CellVGAE shares 99.42% of B cells, 98.75% of CD14 Mono and 96.27% of CD4 T cells with Seurat, with the others having an overlap of slightly over 87% each. The only exception is for Platelets at 78.57%, however, this is due to the extremely small cluster size (CellVGAE correctly finds 11 out of 14 Platelet cells). Overall, CellVGAE achieves an ARI (defined in in Supplementary Information SG) of 0.897 (a perfect match is indicated by an ARI of 1.0) computed using Seurat as the ground truth, while SAM stands at 0.754 and DiffVAE at 0.376. Moreover, for CellVGAE related cell types are close in UMAP space: CD4 T, CD8 T, and NK cells occupy the bottom right, the two monocyte families and DC cells are situated in the top left, whereas the B cells form a separate, distant population in the top right. We recognize that Seurat is a computational method as well and does not represent the ground truth; however, CellVGAE is the only method we tested that can clearly separate the correct number of clusters, and with a high ARI

The *PBMC3k* dataset (10x Genomics) is difficult for SAM, DiffVAE and scVI in terms of cluster definition and separation, as illustrated and discussed in Figure  4. More specifically, none of the existing methods clearly separates the existing clusters, while CellVGAE is able to achieve both tight clusters and definitive separation, as shown by the unsupervised HDBSCAN clustering without any unlabelled cells. SAM is the only method with similarly tight clusters, but it is not capable of separating all cell populations even with its best-performing hyperparameters.

### 3.2 CellVGAE captures pseudo-temporal information in continuous blood cell differentiation

As CellVGAE is capable of representing complex biological signals in difficult scRNA-seq datasets (not exclusively based on transcriptomics profiling, Fig.  3), we now evaluate CellVGAE on a continuous blood cell differentiation dataset consisting of 2730 myeloid progenitors with 3451 genes (Paul *et al.*, 2015), investigating if the model can capture continuous phenotypes. To establish this, it is useful to consider the concept of *pseudotime*, defined as the ordered gradual change in gene expression and serving as a method to place cells along an inferred differentiation trajectory (Ji and Ji, 2019). It was previously shown that neural implementations such as the Deep Count Autoencoder (DCA) (Eraslan *et al.*, 2019) can produce cell embeddings that respect the diffusion pseudotime.

**Fig. 5.**
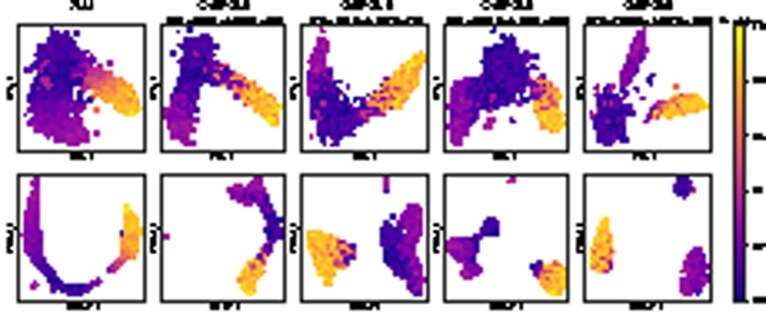
Similarly to other VAEs and SAM, CellVGAE can successfully capture a continuous phenotype such as differentiating blood cells. Illustrated are the cell embeddings computed by SAM and four different configurations of CellVGAE after dimensionality reduction with both PCA and UMAP and with the pseudotime overlay provided by Scanpy (following the workflow suggested by Eraslan *et al.*). The four CellVGAE models use identical settings, including the PKNN graphs, with the exception of the number of HVGs. Compared to other evaluated methods including SAM, DiffVAE and scVI (Supplementary Figs S3 and S4), the clustering behaviour changes with the amount of transcriptomics information provided, leading to a gradual separation of clusters. A similar plot highlighting the discrete cell types identified by Paul *et al.* is provided in Supplementary Figure S5

Like other neural algorithms and SAM, CellVGAE can successfully order the differentiating cells according to the pseudotime (Fig.  5). Differently, however, we notice that as the quantity of transcriptomics information is increased, the clusters become progressively more separated, starting from a single continuous cluster and reaching three clearly delimited clusters. The behaviours of DiffVAE and scVI under the same transcriptomics settings are illustrated in Supplementary Figures S3 and S4, respectively. DiffVAE successfully identifies the single, continuous cluster and can broadly discern two clusters, however not accurately and with no correlation to the amount of transcriptomics information. scVI successfully orders the cells by pseudotime but does not perform any separation. Thus, we recognize that CellVGAE excels at cleanly separating clusters (as in Fig.  2), especially when a large amount of gene expression information is available.

### 3.3 Graph attention coefficients enhance interpretability

We propose two alternative ways of visualizing the learnt attention coefficients (denoted by *α*). To quickly recapitulate, the attention coefficients measure the contribution of each cell to its neighbours in a pairwise manner (Equation 4). Thus, the numerical value associated with two nodes can be interpreted as an edge. For two nodes *i*, *j* the coefficients *α_ij_*, *α_ji_* are different, resulting in a graph that is weighted *and* directed. Each graph attention layer has its own independent set of coefficients.

First, we propose mapping the attention coefficients to a node-level representation by averaging over each node’s neighbourhood, acting as an overlay for two-dimensional projections like UMAP and where the highest resulting values indicate the nodes with the largest contribution in the neural model. Secondly, a finer-grained visualization is enabled by plotting a graph of cells, where the attention coefficients give the edge weights. A simple strategy is to select the top *n* largest coefficients; alternatively and depending on the specific use case, the analysis can exclude certain cell types or focus on a different range of interactions (e.g. the smaller edge weights), providing a variable level of granularity.

**Fig. 6.**
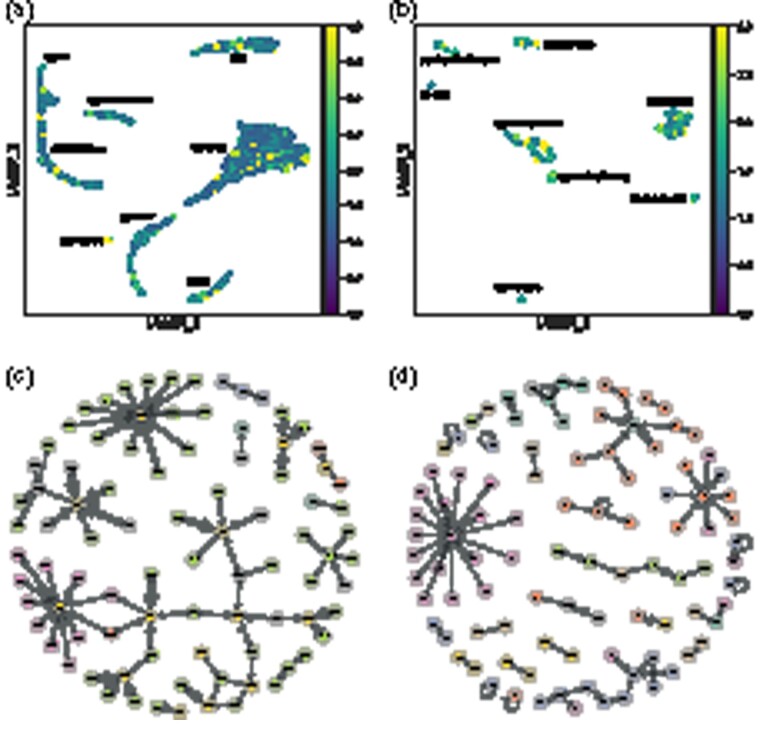
Applying the two interpretable strategies for ‘*PBMC3k*’ (a, c) and ‘*Darmanis*’ (b, d). We chose the ‘*Darmanis*’ dataset since there is a relatively large difference in ARI compared to the other three methods of Table  2, so insights into the model are valuable

We illustrate the two techniques in Figure  6, for two datasets. For both figures and datasets, we chose the second layer of the CellVGAE model and for simplicity have taken the mean across all the attention heads, but the same analysis applies per head and can, in fact, reveal the differences in what each one learns (the individual attention heads are illustrated in Supplementary Information SN). In the standard GAT framework, the attention heads are not enforced to learn different representations, for example by minimizing mutual information, although this could be an interesting future direction.

For *PBMC3k*, a non-trivial finding from the UMAP plot in Figure  6a is that the Platelet cells are highly involved at the level of the second layer. In fact, by inspecting the graph representation (Fig.  6c), we discover that all 14 Platelet cells are among the top contributors, with some of the highest-weight edges (attention coefficients). The successful representation of this dynamic is particularly interesting since the Platelet cluster is by far the smallest, at just over 0.005% of all cells. In Supplementary Information SD and SE we examined whether the high attention coefficients for Platelets arise due to spurious connections in the KNN graph, and compared with PAGA (Wolf *et al.*, 2019), a well-known algorithm for detecting and visualizing connections between cell clusters. The analyses revealed little contribution from the KNN graph structure alone, as well as similar, strong connections identified by PAGA. The other prevalent connections involve CD4 T, CD8 T, and FCGR3A cells. For this particular graph, we notice the existence of long paths and a relatively low number of connected components.

For the *Darmanis* dataset, all 8 cell types are encountered in the relatively heterogeneous graph of Figure  6d, suggesting that multiple different cell types can inform the classification of a single cell. Neurons, which are among the most activated, interact with an Endothelial cell, and another large group, of Fetal quiescent cells, is seen largely interacting with its kind, although it participates in other relationships, mostly with Fetal replicating cells. On this dataset, we notice the presence of self-loops, which might indicate that a cell’s own features are enough to distinguish itself. Overall, the graph is more fragmented (more small, connected components) indicating less cross-talk.

To further our understanding of the attention coefficients, we study if they encode redundant information. More specifically, we selected the top 80 cell pairs from Figure  6c and computed the pairwise Euclidean distances in the 50-dimensional latent space (Supplementary File S2). We find that the overwhelming majority of cell pairs are distant. Thus, high attention coefficients do not correspond to highly similar cells, meaning that they capture an additional layer of information not present in standard VAEs.

### 3.4 Retrieval of marker genes using neural weights analysis

To link the latent dimensions to the genes, the neural weights can be analysed, as initially proposed for DiffVAE. Since CellVGAE uses a simple inner product decoder, we focus on the encoder, a possibility enabled by the use of graph attention layers. Although the mechanism of graph attention layers is different from the simpler dense layers, each GAT layer is parameterized by a learnable weight matrix **W** (Equations 4 and 5). Assuming a CellVGAE architecture with the same number of attention heads, two hidden layers ${\text{GAT}}_{i}^{\left(K\right)}$ with i∈{1,2}, and two layers for the parameters μ and σ: ${\text{GAT}}_{\mu }^{\left(K\right)}$ and ${\text{GAT}}_{\sigma }^{\left(K\right)}$, where *D_i_* is the output dimension of each layer, we can extract the weight matrices $W_{1}\in R^{KD_{1}\times D},\;W_{2}\in R^{KD_{2}\times KD_{1}}$ and $W_{\mu },W_{\sigma }\in R^{L\times KD_{2}}$, where additionally *D* is the input number of dimensions, *L* is the number of latent dimensions and the matrix dimensions follow PyTorch conventions. These allow us to derive the following products for the μ and σ layers:

(12) $$\begin{array}{cc} P_{\mu }=W_{1}^{⊤}\cdot W_{2}^{⊤}\cdot W_{\mu }{}^{⊤} & P_{\sigma }=W_{1}^{⊤}\cdot W_{2}^{⊤}\cdot W_{\sigma }{}^{⊤} \end{array}$$

with dimensions *D *×* L*. The matrices effectively link the genes to the latent dimensions, and an analysis similar to DiffVAE’s can be applied. For each cluster, and then for each latent dimension, the 15 top genes are selected. Overall, the same gene might be selected multiple times, so the number of appearances of genes for all clusters is recorded. We report some of the found genes in descending number of appearances for the first CellVGAE model of Figure  2 (for *S.mansoni*) in Table  1 and additionally, if one gene appears only one time, but is within the top 100 weights in the product matrices, it is included in the bottom row.

**Table 1.** (Top row) Examples of high-weight genes found by CellVGAE in descending order

Matched cluster	*ϵ*	δ′	*μ*
Latent analysis	129510, 191690,	**161920**, **070380**,	**068280**, 169030,
	**070380**, **161920**,	179790, **041550**,	**105220**, **161920**,
	**021340**	**068280**	**070380**, **041550**
Top 100	**142120**, **041540**	**051920**, **158740**	**062490**, **158740**,
			**044680**

*Note*: These genes appear multiple times across latent dimensions (more than 2). (Bottom row) Some genes that appear once but have high values in $P_{\mu }$. Bold genes are also identified as marker genes by SAM. For brevity, we omit the prefix of the full name (e.g. Smp_041540). The three clusters correspond to Figure  2 (leftmost plot).

Out of the genes mentioned in the SAM manuscript, only 5 are in the top 250 variable genes and CellVGAE correctly identifies them. More specifically, the RNA binding protein *nanos-2* (Smp_051920), which is characteristically expressed in δ′ cells, *eled* (Smp_041540) which marks *ϵ* cells, an actin protein (Smp_161920) and *dhand*, a helix-loop-helix transcription factor (Smp_062490) that mark *μ* cells and finally an achaete-scute transcription factor, *astf* (Smp_142120) which is highly enriched in $\epsilon_{\alpha }$ cells, a subpopulation of *ϵ* cells.

### 3.5 CellVGAE representations correspond to known biological signals

We make a first step towards visualizing what the model has learnt in terms of gene expression, starting with the learnt node embeddings, a matrix **E** of dimension *N *×* L*, where *N* is the number of cells and *L* is the latent (output) size, as well as $P_{\mu }$ with dimension *D *×* L*. We select the vector **g** corresponding to a target gene of choice out of $P_{\mu }$ and perform the multiplication E·g, which gives the CellVGAE expression value, used as the hue parameter when plotting. This type of analysis can suggest the contribution of a gene at the level of each cell, providing a granular view of expression within a cluster.

**Fig. 7.**
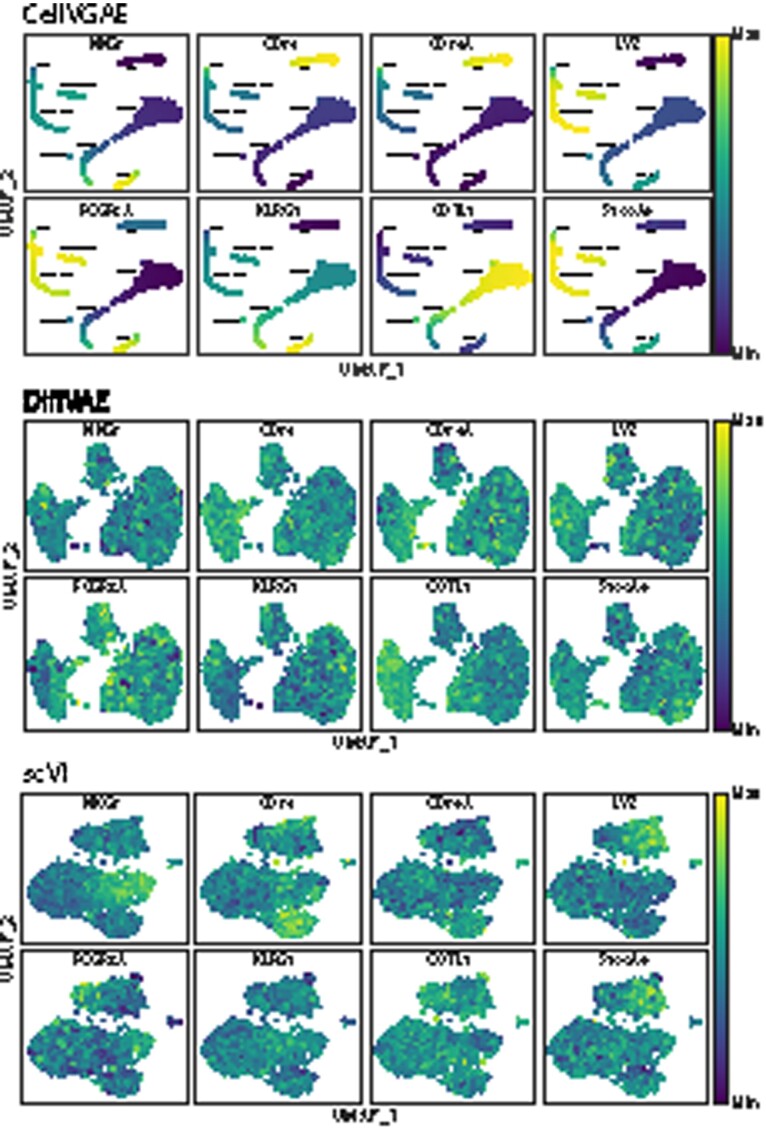
Visualization of learnt model gene expression for eight different genes (NKG7, CD74, CD79A, LYZ, FCGR3A, KLRG1, COTL1, S100A9) from the ‘*PBMC3k*’ dataset for CellVGAE, scVI and DiffVAE. We interpret positive values as a form of high expression and negative values as the opposite

The proposed approach applies to all VAE-based methods and is not specific to CellVGAE. However, as illustrated in Figure  7, only CellVGAE is sensitive enough to visualize the learnt gene expression patterns, confirming that the model captures relevant biological information. Both CellVGAE and scVI successfully represent clusters enriched for some typical marker genes, such as NKG7, FCGR3A, and LYZ, although the tight cluster formations of CellVGAE aid the visualization. However, other genes lead to much more diffuse scVI representations, for example KLRG1, which should be expressed in NK cells (Wang *et al.*, 2013), or CD74 which should be enriched in B cells (Naeim *et al.*, 2008). DiffVAE does not convincingly capture gene-level information in any of the proposed examples.

### 3.6 Cell clustering performance on 10 well-annotated datasets

Next, we present an extensive evaluation of CellVGAE, SAM, DiffVAE and scVI on nine challenging and well-annotated scRNA-seq datasets (covering a wide range on the network sensitivity spectrum, Supplementary Information SA) where we show improved clustering performance, as well as on the RETINA dataset of 26.5K cells (Shekhar *et al.*, 2016). Dataset availability and other characteristics are provided in Supplementary Table S6.

All 9 datasets were used to benchmark SAM alongside Seurat, SC3 (Kiselev *et al.*, 2017) and SIMLR in (Tarashansky *et al.*, 2019). Here, we do not reproduce the evaluation for the other algorithms. RETINA is included as it is the largest stand-alone dataset used to evaluate scVI in its publication. We run all four algorithms ourselves and report the results in Table  2. The main metric used to evaluate clustering performance is the adjusted rand index (ARI), a measure of the similarity between two data clusters. It is the standard choice for this type of evaluation and it is defined mathematically in Supplementary Information SG. We also include the silhouette coefficient (SC), similarly defined in Supplementary Information SG. Although we prioritize the ARI as a metric of clustering performance, the SC is a popular metric that penalizes clusters that are more spread out or not sufficiently delimited. We also keep track of the number of clusters found by each method, compared to the ground truth number (Supplementary Information SK).

**Table 2.** Clustering results on the 10 datasets (listed in Supplementary Table S6)

		SAM		CellVGAE			DiffVAE			scVI	
Dataset	No. of cells	ARI	SC	ARI	SC	N	ARI	SC	N	ARI	SC
Darmanis	420	0.9199	0.7008	**0.9472**	0.7724	0	0.8621	0.7104	4	0.8105	0.4483
Wang	457	0.8944	0.2932	**0.9418**	0.4772	1	0.9213	0.5939	5	N/A	
Baron1	1937	0.9625	0.7084	**0.9670**	0.7476	0	0.9659	0.6953	27	0.9592	0.6000
Baron2	1724	0.9681	0.6447	**0.9817**	0.7139	0	0.9428	0.6926	6	0.9688	0.5199
Baron3	3605	0.9456	0.7256	**0.9723**	0.7376	0	0.9459	0.6626	56	0.9628	0.5918
Baron4	1303	0.9301	0.7588	**0.9764**	0.7474	0	0.9287	0.7849	0	0.9238	0.6225
Loh	498	0.9674	0.7434	**0.9684**	0.8187	0	0.9560	0.7503	8	N/A	
Segerstolpe	2209	0.9360	0.3843	**0.9734**	0.6834	0	0.9667	0.7019	0	0.9330	0.6167
Muraro	2126	0.9262	0.7329	**0.9503**	0.7272	0	0.9496	0.8556	0	0.9188	0.6989
RETINA	26 439	0.9695	0.8084	0.9699	0.6082	17	0.9778	0.7391	533	0.9664	0.5682

*Note*: The metrics used are the adjusted rand index (**ARI**), silhouette coefficient (**SC**) and noise (**N**). Noise represents the number of cells that cannot be assigned to a cluster according to the HDBSCAN clustering algorithm used for CellVGAE and DiffVAE. For CellVGAE, in certain datasets with many small clusters, i.e. ‘*Baron1*’, ‘*Baron2*’, it is possible to manually separate more (extremely small) clusters that were missed by the automatic clustering procedure for additional performance. scVI is not benchmarked for datasets that do not provide raw counts. CellVGAE uses KNN/PKNN graphs with k=5 and the KL loss. We notice that DiffVAE performs better on the larger rather than smaller datasets, which is expected for dense neural networks. Bold values represent the highest ARI scores for each dataset.

Finally, for CellVGAE and DiffVAE we report the number of cells that were not clustered by HDBSCAN, which we call noise (N). This does not apply to SAM as it internally applies a further clustering step to HDBSCAN outliers using KNN classification (details of the implementation are available in Tarashansky *et al.* (2019) and the SAM source code), and neither to scVI as the internal clustering procedure does not rely on HDBSCAN. We do not apply further clustering for the results of Table  2, but we employ a post-processing step to ensure that very small clusters are not missed by HDBSCAN. Our procedure is detailed in Supplementary Information SK.

CellVGAE obtains the highest ARI score on every one of the nine challenging (according to the network sensitivity metric) datasets, some being very close to the competition (e.g. on *Muraro*, *Baron1*), whereas others such as *Darmanis*, *Wang* and *Baron3* exhibit considerably larger scores.

Generally, all methods achieve silhouette scores that can be considered good (≥0.65), a few exceptions being some very low scores for SAM. To better understand CellVGAE’s silhouette scores, we provide illustrations of the CellVGAE and SAM clusters for the 9 datasets in Supplementary Figure S7a–i. Compared to the clusters found by the existing VAE methods (for example Fig.  4), the techniques that exploit cell connectivity (CellVGAE and SAM) tend to form more complicated shapes when visualized in two dimensions using UMAP. In particular, one pattern common to both CellVGAE and SAM is the presence of clusters that are broadly linear in shape (SAM in Supplementary Fig. S7a, both in Supplementary Fig. S7c, CellVGAE in Supplementary Fig. S7g, SAM in Supplementary Fig. S7i and SAM in Fig.  5.). Differently from SAM, CellVGAE identifies clusters with topology involving holes and arcs, such as the Beta and Alpha cells from the *Muraro* dataset (Supplementary Fig. S7i), the Alpha cells from the *Segerstolpe* dataset (Supplementary Fig. S7h) and similar instances in Supplementary Figure S7f and d.

All methods perform exceptionally well on RETINA (Shekhar *et al.*, 2016) thanks to the large number of cells. The reference RETINA clusters are derived computationally using Louvain clustering, thus not necessarily corresponding to the ground truth. DiffVAE achieves the highest ARI, however by discarding 533 cells that are unclassifiable by HDBSCAN due to the very spread out clusters. On the other hand, we again confirm that CellVGAE forces tight and well-separated cluster formations by only reporting 17 unclustered cells. By visualizing the found clusters (Supplementary Fig. S7j), we observe that CellVGAE separates a cluster with mixed reference cell types, that differentially expresses Calm1, Scg2, Trpm1 and Scgn (Scanpy’s rank_genes_groups() with the ‘t-test’ method). Discarding this cluster (an additional 508 cells), we arrive at an ARI of 0.9775 and an SC of 0.7670, i.e. essentially matching DiffVAE’s ARI.

To complete the analysis, we provide an extensive evaluation of CellVGAE on two additional values of k, k=3 and k=10 (Table  2 uses k=5), using GAT (default for CellVGAE), GCN and GATv2 layers (Supplementary Table S4). We also experimented with an additional decoder component in the form of a dense neural network that reconstructs the gene expression values, as in existing VAE methods, with results reported in Supplementary Table S5. Overall, we find that the other graph settings perform slightly worse but still improve over existing methods. The addition of the decoder neural network does not lead to considerable differences.

### 3.7 Training time and scalability

With the introduction of CellVGAE, we diverge from existing VAE implementations by employing graph convolutions and raise new questions about the scalability of the method for high-throughput sequencing datasets. An important difference is that CellVGAE uses a single graph that is fully loaded into memory throughout training (the impact in terms of memory utilization is minimal), avoiding the need to mini-batch the data.

**Fig. 8.**
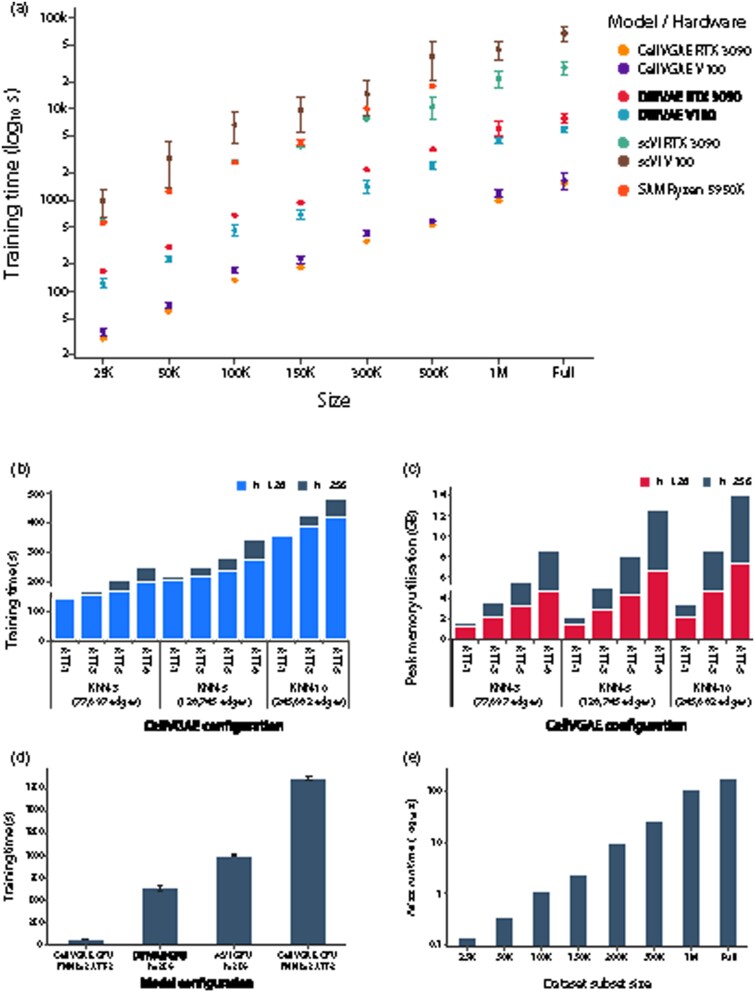
Training characteristics such as time and peak memory consumption for CellVGAE and existing methods on different hardware platforms. Unless otherwise specified, the platform equipped with the AMD Ryzen 5950X processor and the NVIDIA RTX 3090 GPU represents the default choice for CPU and GPU experiments. (**a**) Training times for CellVGAE, DiffVAE, scVI and SAM on subsets of the 1.3 million cells dataset, where ‘K’ denotes thousands and ‘M’ millions. The neural models were trained for 200 epochs on two different GPU platforms: an NVIDIA RTX 3090 (24GB VRAM) and an NVIDIA V100 (32GB VRAM). SAM is exclusive to CPUs and was trained on an AMD Ryzen 5950X for 200 iterations. All neural models were trained with identical (or equivalent) hyperparameters, except for the ‘1M’ and ‘Full’ datasets where CellVGAE required slightly downscaled parameters to fit in 24GB VRAM (Supplementary Information SL). SAM results for ‘1M’ and ‘Full’ are unavailable and thus not displayed. (**b**) Training time for the CellVGAE GPU models (ATT, number of attention heads; h, hidden dimensions). (**c**) Peak memory consumption for the CellVGAE GPU models (ATT, number of attention heads; h, hidden dimensions). (**d**) CellVGAE training times on both GPUs and CPUs. (**e**) Execution time of exact graph generation with Faiss (GPU)

We analysed the training time for eight random subsets of an open-source scRNA-seq dataset of 1.3 million mouse brain cells (from 10x Genomics). Combined with the fast GPU implementation of GNNs provided by PyTorch Geometric, CellVGAE offers between ×3.5 to ×21 faster training times than DiffVAE and scVI, depending on the subset and hardware, as illustrated in Figure  8a. At the same time, CellVGAE GPU is between ×18.5 to ×33.5 faster than SAM, a CPU-exclusive algorithm. SAM failed to run for the ‘1M’ and ‘Full’ subsets, limiting its applicability to large-scale datasets.

We further study the training time and resource utilization of CellVGAE with several hyperparameters (Fig.  8b and c) on RETINA. The training time increases sublinearly in the number of graph edges and the number of attention heads (Fig.  8b). Similarly, doubling the hidden layer size produces only minimal increases in training time. On the other hand, the peak memory consumption grows more abruptly with the number of attention heads, increasing roughly linearly when doubling the hidden layer size (Fig.  8c). This is expected, as the parallel attention mechanism stores and updates separate copies of all the attention coefficients. We thus see that CellVGAE offers a trade-off between low training times and high memory usage.

As a finer-grained comparison, we also discovered that the training time of CellVGAE on a CPU is on the same order of magnitude as existing VAE models trained on GPUs (Fig.  8d, on RETINA), enabling the possibility to train large models when video memory is insufficient. Finally, we benchmarked the extremely efficient similarity search library Faiss (Johnson *et al.*, 2017), an optional dependency of the CellVGAE implementation, for KNN graph generation on the same subsets of up to 1.3 million cells (Fig.  8e).

Our analysis using the PyTorch profiler reveals that the KNN graph itself occupies a negligible amount of memory, with occasional attention-related operations (e.g. concatenation, index select) greatly contributing to the peak consumption. As Faiss is GPU-enabled, it can perform exact KNN computations in under 5 min for all eight subsets, with the possibility to scale to billions of vectors with approximative algorithms.

## 4 Conclusion

We introduced CellVGAE, a machine learning architecture that integrates the benefits of graph-based clustering techniques with recent advancements in neural networks. We showed that CellVGAE performs consistently well in finding accurate, informative clusters, even when applied to complex datasets with subtle signals or when modelling properties not directly related to transcriptomics. The method produced excellent results on nine challenging datasets in terms of the most relevant clustering metrics. Furthermore, we delivered three strategies for interpretability: (i) high-weight gene identification, useful for gene markers and gene set enrichment analysis; (ii) visualization of learnt expression, per-gene; and (iii) a practical interpretation of the attention coefficients. Overall, we showed that the combined use of neural networks and graphs is effective for scRNA-seq data analysis. Lastly, by examining the training time and resource utilization, we concluded that CellVGAE is several times faster than existing VAE implementations on equivalent hardware.

## Supplementary Material

btab804_Supplementary_Data

## Data Availability

The data underlying this article are available in the article and in its online supplementary material.
