# Supplementary material for: CellVGAE: an unsupervised scRNA-seq analysis workflow with graph attention networks
Source: Bioinformatics. 2021 Dec 2;38(5):1277–86. doi: 10.1093/bioinformatics/btab804 (PMC8825872; doi:10.1093/bioinformatics/btab804)
Supplement: btab804_Supplementary_Data [file btab804_supplementary_data.zip › Supplementary Information.pdf]

Supplementary Information

A Network sensitivity

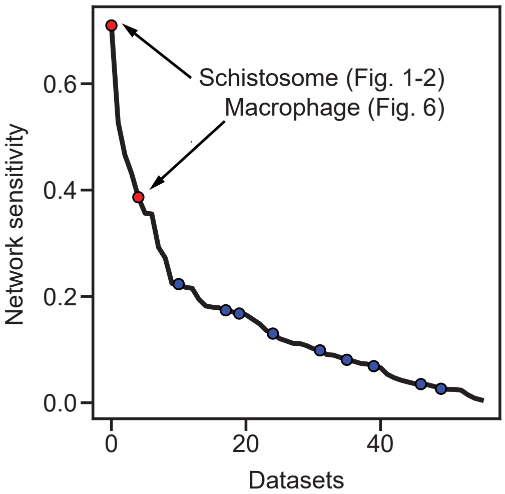

Supplementary Figure 1: Exact reproduction of Figure 4 from the SAM manuscript [12], depicting 56 datasets ranked by network sensitivity in descending order. The *Schistosoma mansoni* dataset has the largest sensitivity by far, while the *Macrophages* dataset is itself remarkably difficult compared to the majority of other datasets. The other 9 datasets included in our analysis (Table 2) are pictured using blue dots.

B DiffVAE models applied to the *Macrophages* dataset

DiffVAE was configured to use two hidden layers of size 256, a latent dimension of 50, a batch size of 128, and the default learning rate of 0.001. The number of input HVGs is selected from the set $\{100, 150, 250, 500, 750, 1{,}000, 2{,}500, 5{,}000\}$. The last two configurations were trained for 200 epochs, while the rest used 100 epochs. The corresponding UMAP plots are presented in Supplementary Figure 2.

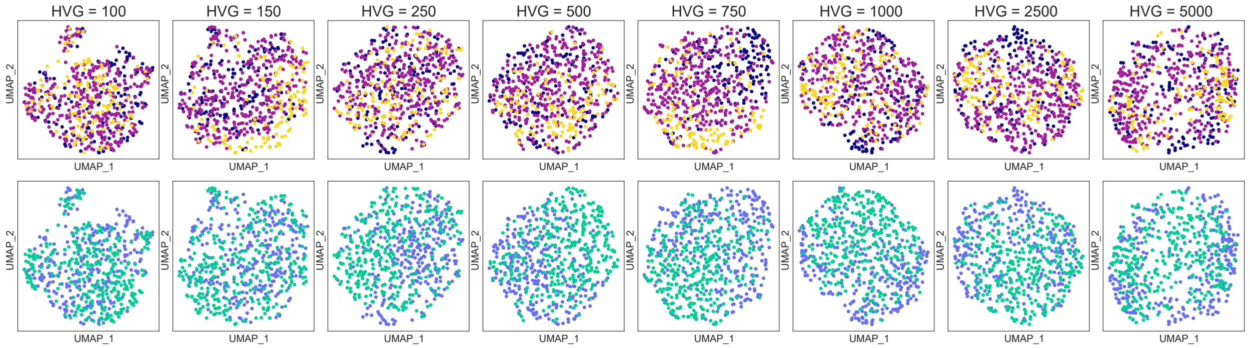

Supplementary Figure 2

C DiffVAE and scVI pseudotime analysis on continuous blood cell differentiation

DiffVAE was configured to use two hidden layers of size 256, a latent dimension of 50, a batch size of 128, and the default learning rate of 0.001. The number of input HVGs is selected from the set $\{500, 750, 1{,}000, 2{,}500\}$. The models were trained for 200 epochs. The corresponding PCA and UMAP plots are presented in Supplementary Figure 3.

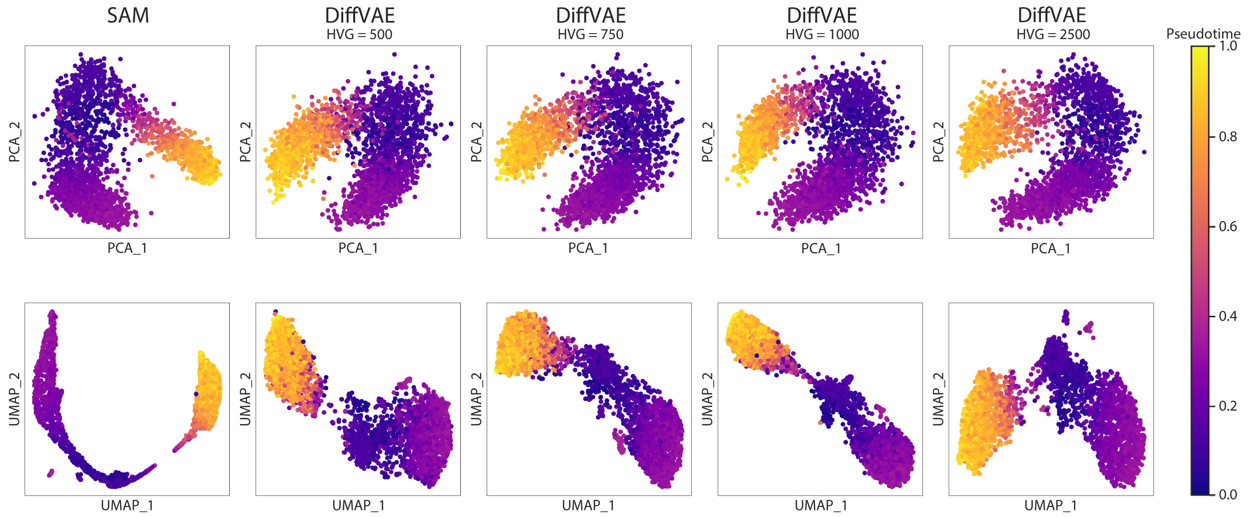

Supplementary Figure 3: PCA and UMAP plots of DiffVAE using varying amounts of transcriptomics information for the myeloid progenitors dataset. SAM is also pictured for comparison.

scVI used default settings with two hidden layers of size 256 and 50 latent dimensions. The model was trained for 200 epochs.

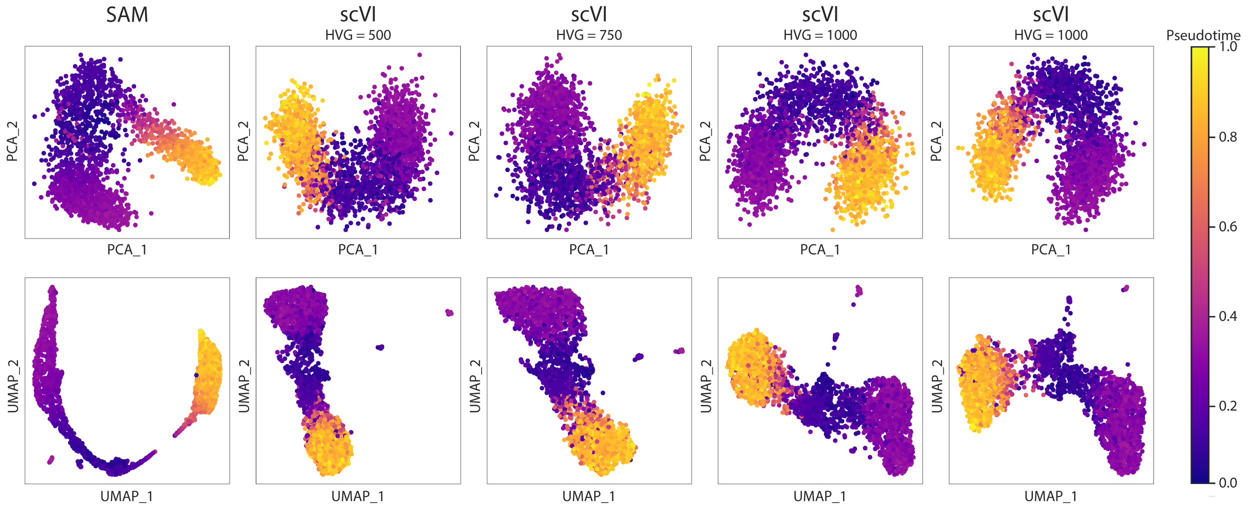

Supplementary Figure 4: PCA and UMAP plots of scVI using varying amounts of transcriptomics information for the myeloid progenitors dataset. SAM is also pictured for comparison.

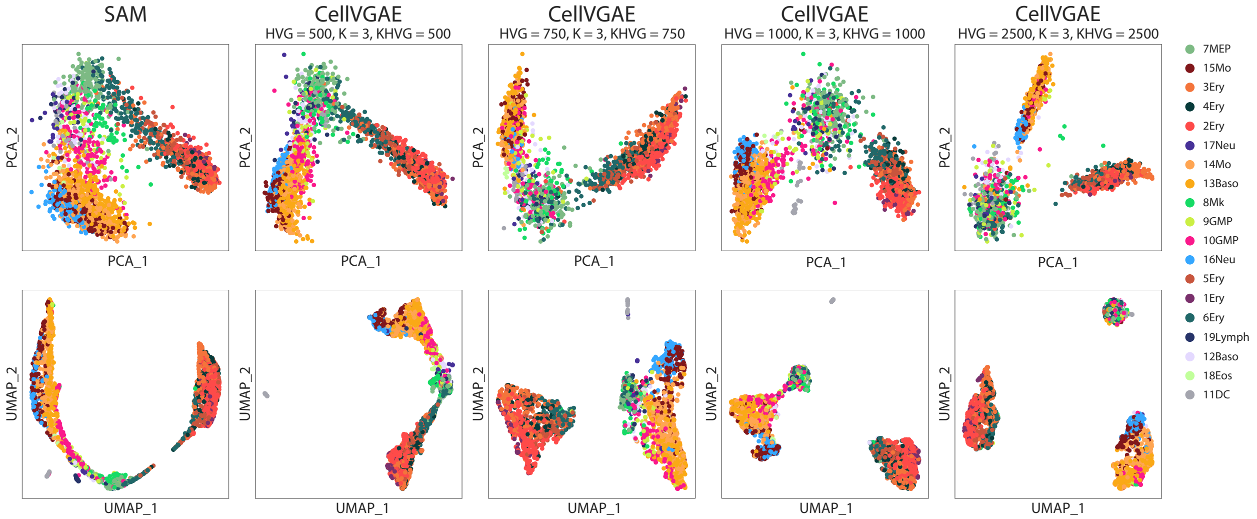

Supplementary Figure 5: Same PCA and UMAP plots as Figure 5 but highlighting the discrete cell types.

D *PBMC3k* platelet connectivities

To investigate whether the high attention coefficients attributed to the Platelet cell type by CellVGAE are caused by spurious connections in the KNN graph (we used $k = 20$), we first selected cells with large amounts of inter-cluster connections. After this step, we arrived at 232 cells with counts of their intra-cluster and inter-cluster connections, also available as fractions for each cell type (for each cell, we divided the number of edges connecting it to other clusters by the total number of connections). These numbers are available as different sheets in Supplementary File 1 (spreadsheet, `.xlsx` format). The cells are numbered by the order in which they appear in the HVG input file (we used 250 HVGs). The counts do not include self-connections. The analysis and numbers presented in this section are based on Supplementary File 1, and the code is available in the repository.

First of all, the KNN graph generation step can produce cells with a number of connections that is not necessarily equal to k. For example, the `buildKNNGraph()` function from the `scran` package [37] allows more edges per cell: in our case more than 20 connections, but we did not encounter cells with less than k connections. In contrast, the CellVGAE KNN and PKNN functions allow the user to set thresholds on the closeness of cells (based on the used distance metric), such that distant connections are not included and the number of connections per cell can be lower than k.

This analysis reveals that the Platelet cells are generally highly connected to other Platelet cells (intra-cluster). On average, 59.82% of connections are to other Platelets, with only 2 cells having 35.48% and 52.17% connections to other Platelets, and the others with $\geq 60\%$ connectivity to

Platelets. Inter-cluster connections are generally to CD8 T and CD4 T cells, with only two Platelets connected to the CD14+ Mono and FCGR3A+ Mono clusters.

In contrast, other cells exhibit strong inter-cluster connections, such as NK cells that are generally well-connected to CD8 T and CD4 T cells in addition to intra-cluster edges to other NK cells, as well as the CD14+ Mono, FCGR3A+ Mono and DC cells that are similarly connected. In addition, cells such as the two T types and the two Mono types often exhibit more than 20 connections, up to 149, although that is an exception and the average number of connections is 31.026. All but two Platelet cells have 20 connections, with the other two having 23 and 31 connections.

On the whole, Platelet cells are not particularly well represented in the KNN graphs, making it very difficult to establish that the high attention coefficients are caused by spurious connections or heavy biases.

E *PBMC3k* analysis with PAGA

To further investigate the validity of the high attention coefficients of Platelet cells in Figure 6, we perform three different experiments using Partition-based graph abstraction (PAGA). More specifically, the first two experiments involve default PAGA settings on the preprocessed *PBMC3k* dataset, available through the Scanpy function `scanpy.datasets.pbmc3k_processed()`. First, we run the algorithm using the default model version (v1.2), visualised in Supplementary Figure 6a and then using v1.0 in Supplementary Figure 6b.

The code preceding PAGA is reproduced below:

```
adata = sc.datasets.pbmc3k_processed()
sc.tl.pca(adata, svd_solver='arpack')
sc.tl.diffmap(adata)
sc.pp.neighbors(adata, n_neighbors=20, use_rep='X_diffmap')
```

where we used 20 neighbours as CellVGAE used a KNN graph with $k = 20$ for this dataset.

From the visualisations in Supplementary Figure 6a and Supplementary Figure 6b it is clear that PAGA indicates high-confidence connections between the Platelet cluster and other partitions, especially the FCGR3A+ Mono cells, but also CD14+ Mono and CD8 T cells. This is confirmed by the confidence values in Supplementary Table 1. PAGA confidence '*should be interpreted as the ratio of the actual versus the expected value of connections*

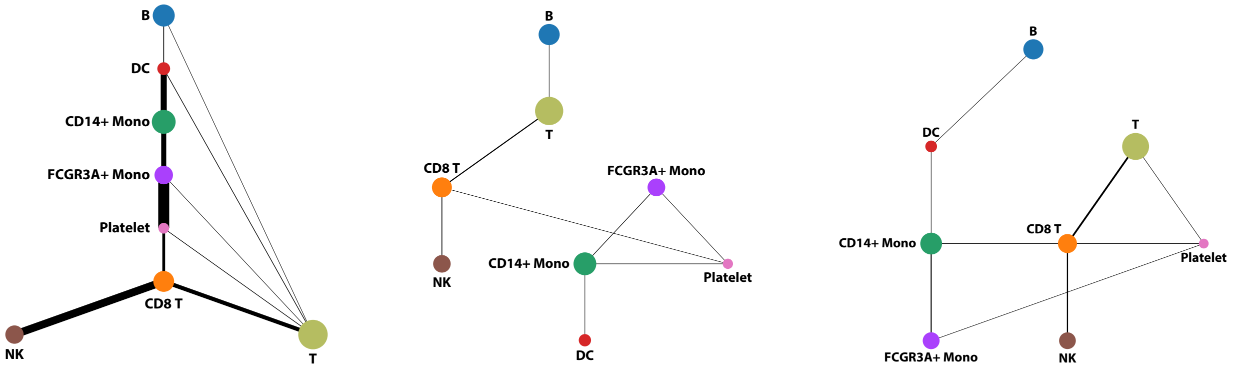

(a) PAGA v1.2 **(b)** PAGA v1.0 **(c)** PAGA with attention (v1.0)

Supplementary Figure 6: Different configurations of PAGA for the *PBMC3k* dataset.

under the null model of randomly connecting partitions' (quote from the source code: https://github.com/theislab/scanpy/blob/master/scanpy/tools/_paga.py#L35). Supplementary Figure 6b and Supplementary Table 2 indicate the same trends, but less pronounced.

	B	CD8 T	CD14+	DC	FCGR3A+	NK	Platelet	T
B	0.000	0.002	0.000	0.069	0.052	0.000	**0.000**	0.026
CD8 T	0.002	0.000	0.001	0.008	0.003	0.705	**0.256**	0.370
CD14+	0.000	0.001	0.000	0.551	0.458	0.000	**0.330**	0.004
DC	0.069	0.008	0.551	0.000	0.000	0.000	**0.000**	0.062
FCGR3A+	0.052	0.003	0.458	0.000	0.000	0.000	**1.000**	0.028
NK	0.000	0.705	0.000	0.000	0.000	0.000	**0.000**	0.007
Platelet	**0.000**	**0.256**	**0.330**	**0.000**	**1.000**	**0.000**	**0.000**	**0.059**
T	0.026	0.370	0.004	0.062	0.028	0.007	**0.059**	0.000

Supplementary Table 1: Confidence values for PAGA v1.2. Platelets are highlighted in bold.

	B	CD8 T	CD14+	DC	FCGR3A+	NK	Platelet	T
B	0.000	0.000	0.000	0.005	0.006	0.000	**0.000**	0.010
CD8 T	0.000	0.000	0.000	0.001	0.000	0.076	**0.011**	0.104
CD14+	0.000	0.000	0.000	0.032	0.059	0.000	**0.019**	0.002
DC	0.005	0.001	0.032	0.000	0.000	0.000	**0.000**	0.008
FCGR3A+	0.006	0.000	0.059	0.000	0.000	0.000	**0.041**	0.008
NK	0.000	0.076	0.000	0.000	0.000	0.000	**0.000**	0.002
Platelet	**0.000**	**0.011**	**0.019**	**0.000**	**0.041**	**0.000**	**0.000**	**0.005**
T	0.010	0.104	0.002	0.008	0.008	0.002	**0.005**	0.000

Supplementary Table 2: Confidence values for PAGA v1.0. Platelets are highlighted in bold.

	B	CD8 T	CD14+	DC	FCGR3A+	NK	Platelet	T
B	0.000	0.002	0.000	0.020	0.000	0.000	**0.000**	0.006
CD8 T	0.002	0.000	0.000	0.000	0.000	0.118	**0.020**	0.179
CD14+	0.000	0.000	0.000	0.052	0.105	0.000	**0.012**	0.000
DC	0.020	0.000	0.052	0.000	0.002	0.000	**0.000**	0.001
FCGR3A+	0.000	0.000	0.105	0.002	0.000	0.000	**0.012**	0.000
NK	0.000	0.118	0.000	0.000	0.000	0.000	**0.000**	0.009
Platelet	**0.000**	**0.020**	**0.012**	**0.000**	**0.012**	**0.000**	**0.000**	**0.027**
T	0.006	0.179	0.000	0.001	0.000	0.009	**0.027**	0.000

Supplementary Table 3: Connectivities values for PAGA v1.0 with attention. Platelets are highlighted in bold.

For the last experiment, we performed the same analysis but replaced the `connectivities` variable computed by `sc.pp.neighbors()` with the attention coefficients computed by CellVGAE, an array with $86{,}251$ entries (Supplementary Figure 6c and Supplementary Table 3). We used PAGA v1.0 as its computation is based on the `connectivities` instead of `distances` (as in v1.2), which are left unchanged. We obtain very similar representations to PAGA v1.0 (Supplementary Figure 6b and Supplementary Table 2), meaning that the attention coefficients largely capture the same inter-cluster relationships.

F CellVGAE and SAM plots for the 9 benchmarks datasets

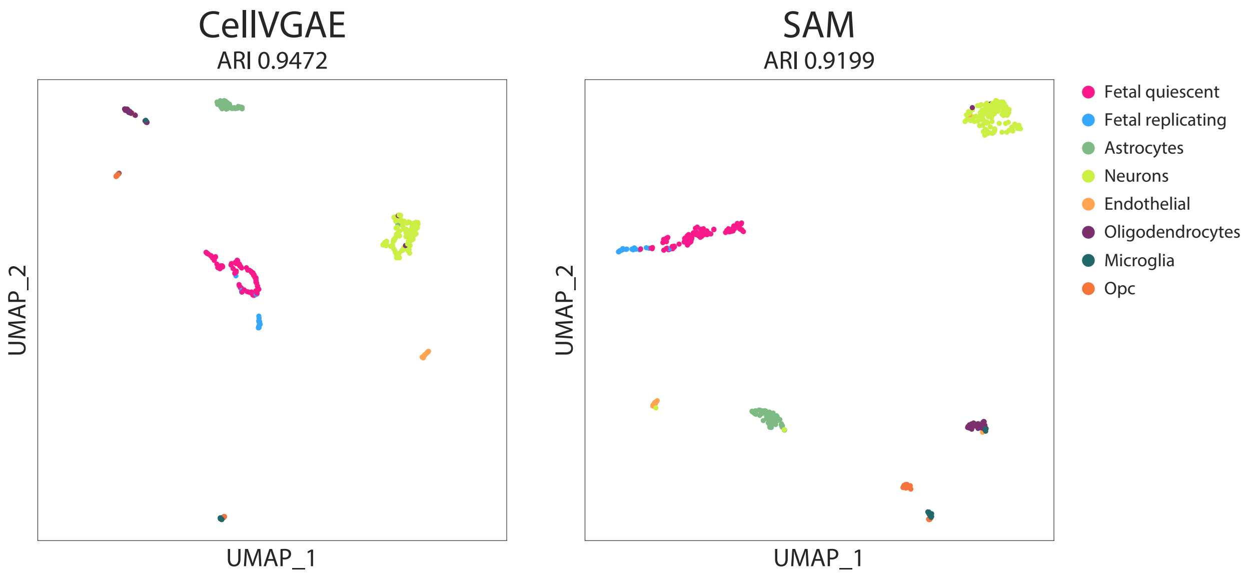

(a) UMAP plots of CellVGAE and SAM for the *Darmanis* dataset. CellVGAE used 250 HVGs and a KNN graph with $k = 5$ based on the original high-dimensional gene expression values with $d = 100$.

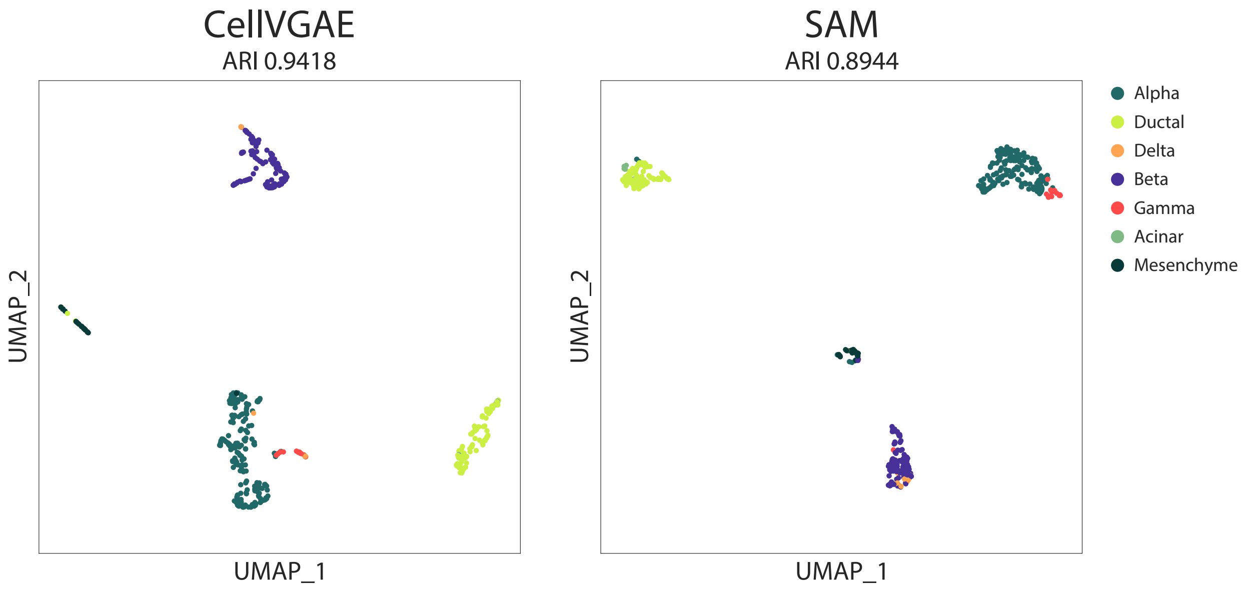

(b) UMAP plots of CellVGAE and SAM for the *Wang* dataset. CellVGAE used 250 HVGs and a KNN graph with $k = 5$ and $KHVG = 250$.

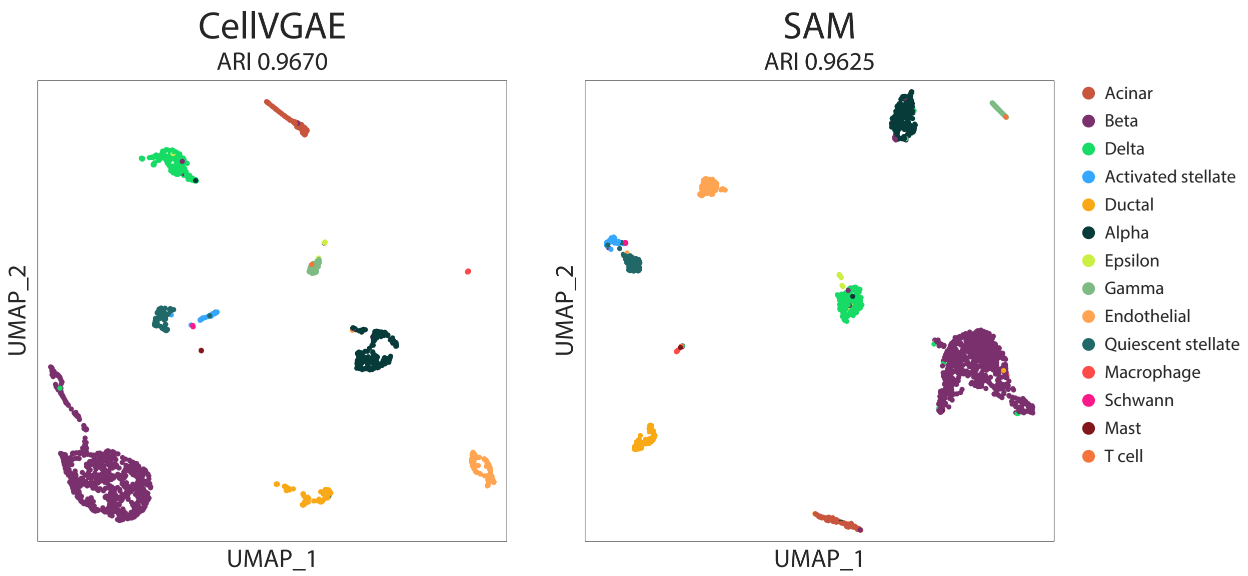

(c) UMAP plots of CellVGAE and SAM for the *Baron1* dataset. CellVGAE used 500 HVGs and a PKNN graph with k $=5$, KHVG $=1{,}000$.

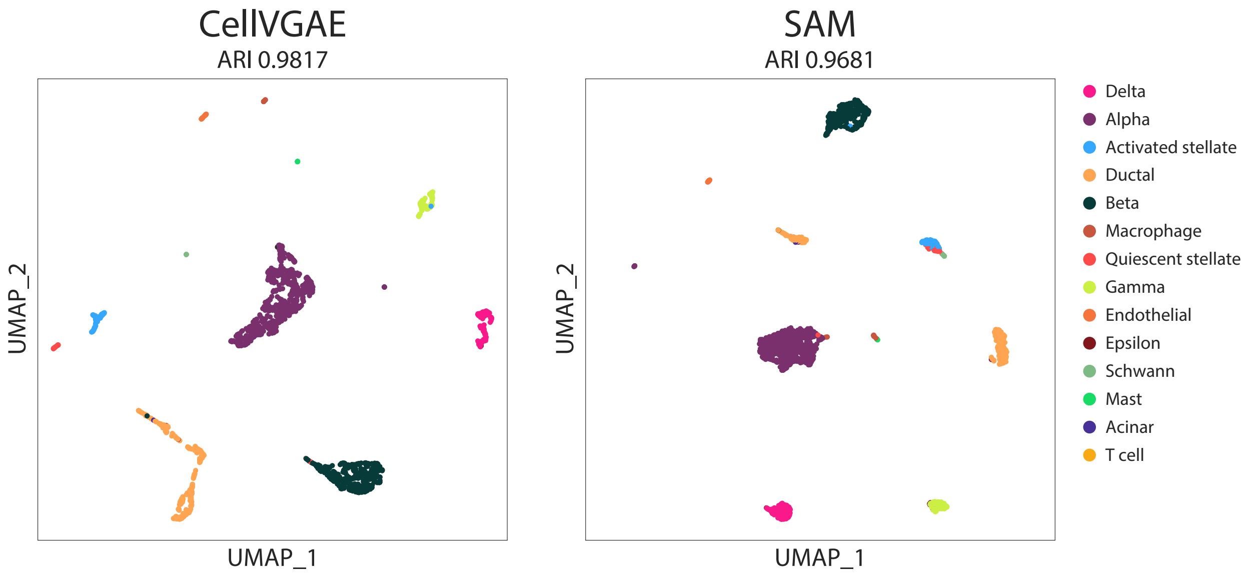

(d) UMAP plots of CellVGAE and SAM for the *Baron2* dataset. CellVGAE used 500 HVGs and a KNN graph with k $=5$, KHVG $=500$, and d $=50$.

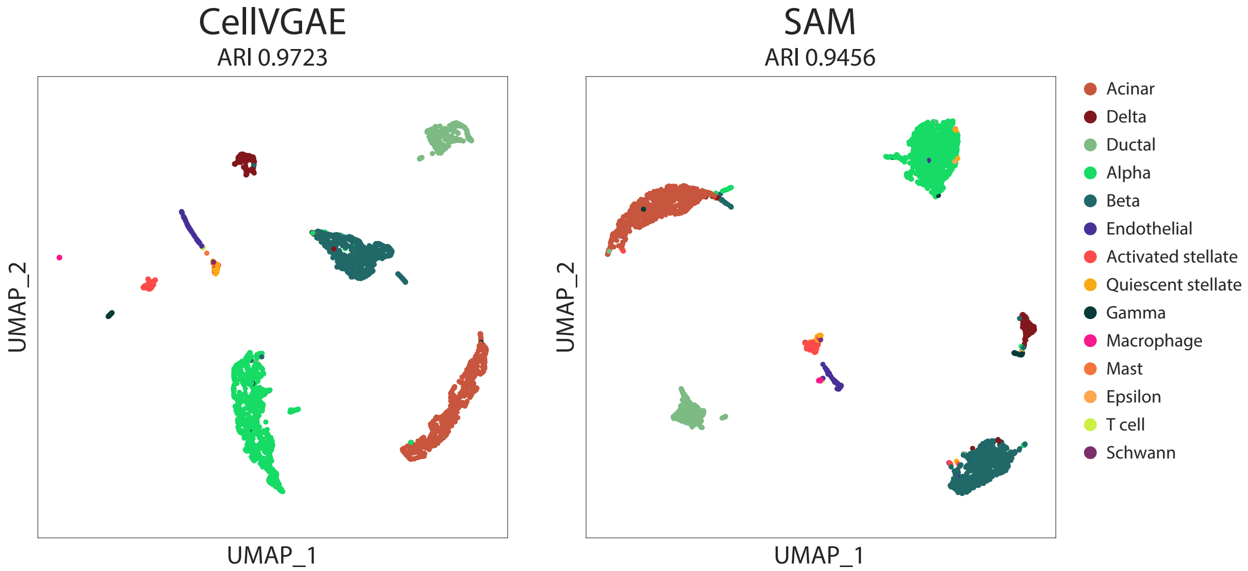

(e) UMAP plots of CellVGAE and SAM for the *Baron3* dataset. CellVGAE used 500 HVGs and a KNN graph with k = 5, KHVG = 500, and d = 50.

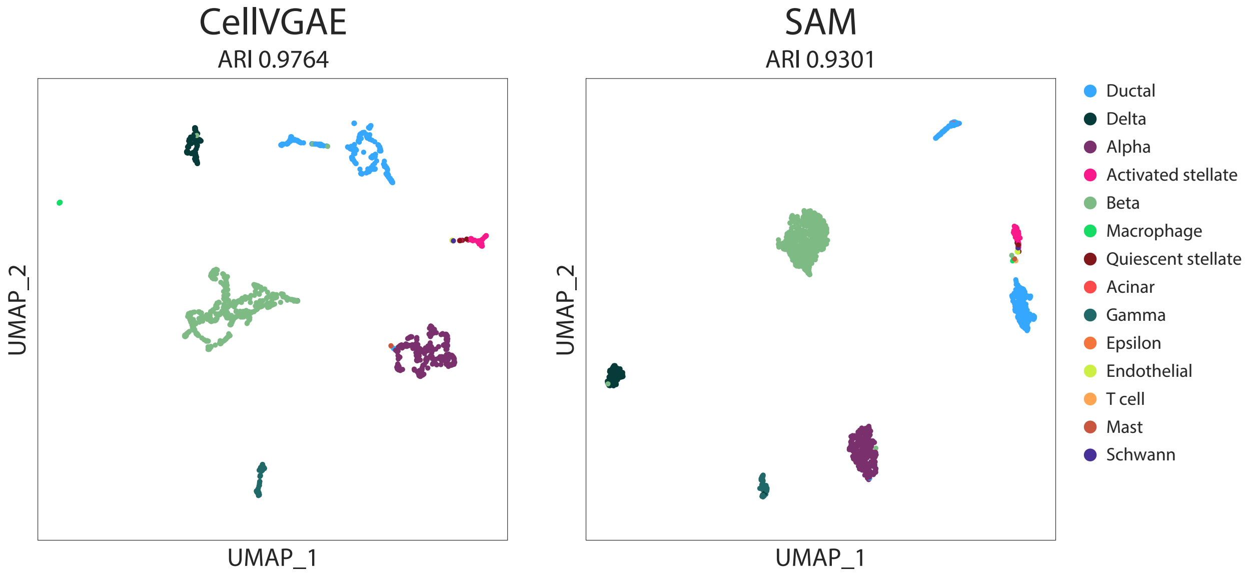

(f) UMAP plots of CellVGAE and SAM for the *Baron4* dataset. CellVGAE used 250 HVGs and a KNN graph with k = 5, KHVG = 250, and d = 10.

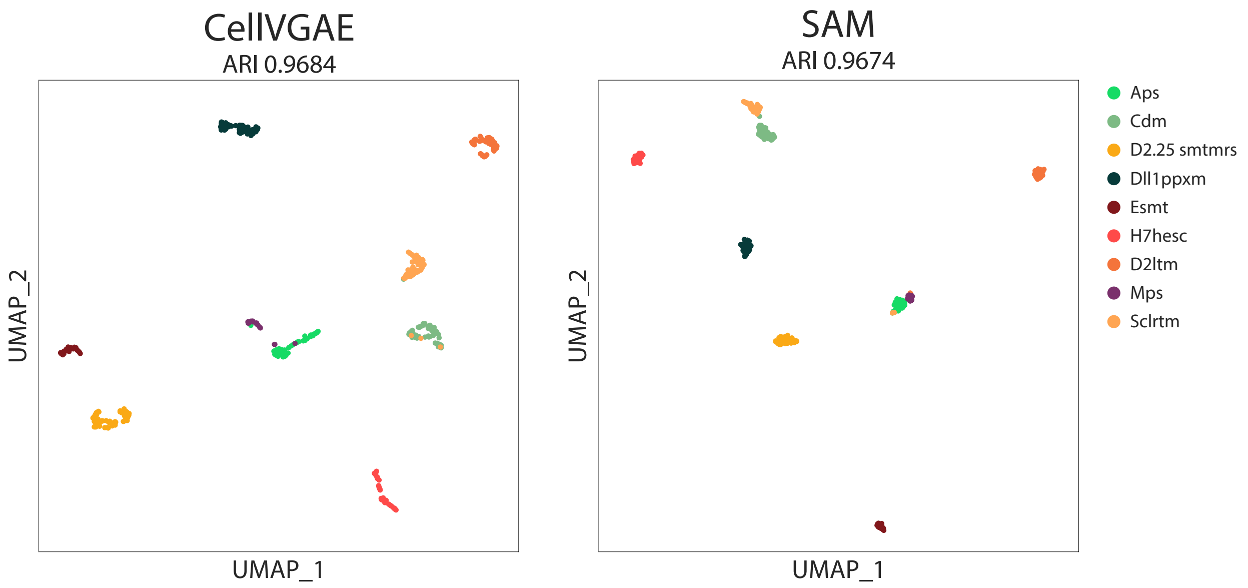

(g) UMAP plots of CellVGAE and SAM for the *Loh* dataset. CellVGAE used 500 HVGs and a KNN graph with k = 5, KHVG = 500, and d = 50.

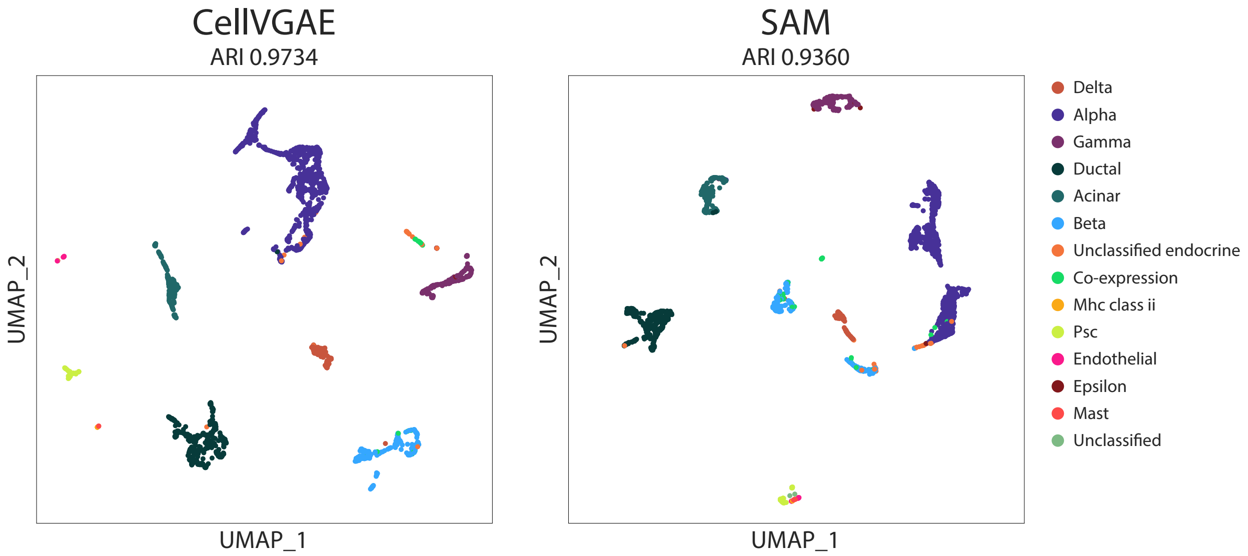

(h) UMAP plots of CellVGAE and SAM for the *Segerstolpe* dataset. CellVGAE used 500 HVGs and a PKNN graph with k = 5, KHVG = 500.

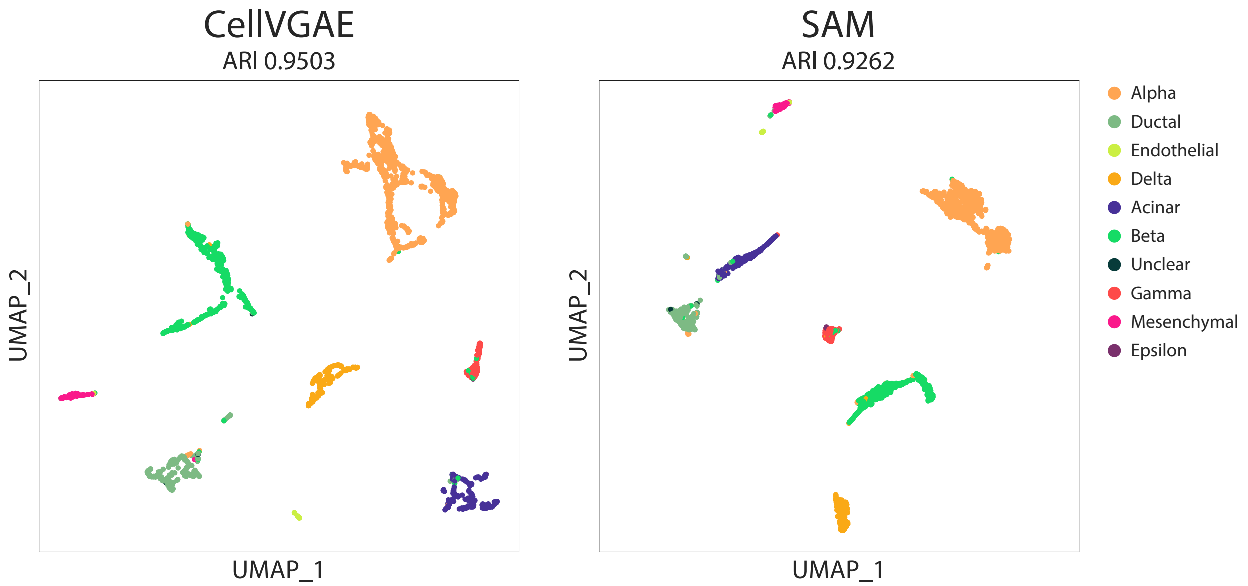

(i) UMAP plots of CellVGAE and SAM for the *Muraro* dataset. CellVGAE used 500 HVGs and a KNN graph with $\mathrm{k} = 5$, $\mathrm{KHVG} = 500$.

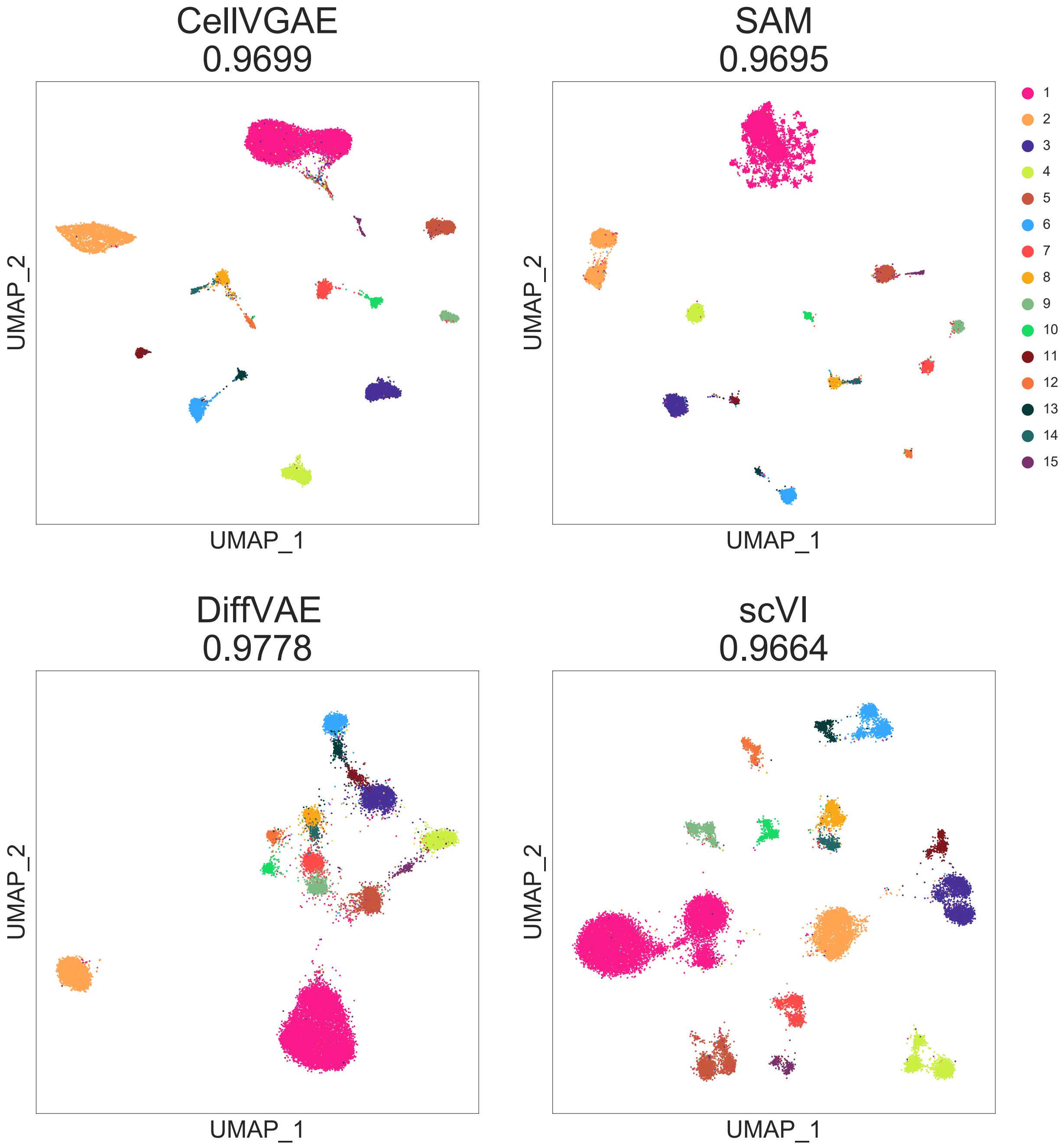

(j) UMAP plots of CellVGAE, SAM, DiffVAE and scVI for the RETINA dataset. CellVGAE used 1,000 HVGs and a PKNN graph with k = 5, KHVG = 1000, and d = 50. DiffVAE and scVI used the top 1,200 HVGs.

Supplementary Figure 7: Visualisation of the learnt representations and HDBSCAN clusters for the 9 benchmarks datasets and the RETINA dataset from Table 2. All CellVGAE models used two layers with a hidden dimension of 128, 50 latent dimensions, and a learning rate of 0.0001. The specifics of each model are provided in the corresponding subfigure.

We plot the UMAP representations for RETINA using the ground truth colouring in Supplementary Figure 7j. CellVGAE forces a cluster of mixed cell types just below the largest (pink) cluster and since this is relatively compact it is recognised by HDBSCAN as a separate cluster, thus reducing the ARI score. Notice that the existing VAE methods, especially DiffVAE, produce very spread out clusters which are problematic for sensitive clustering algorithms such as HDBSCAN. For scVI, the largest cluster is reported as split in two, so all calculations are performed on the merged cluster. Finally, SAM provides tight and well-separated clusters as well, although the largest one has a very grainy structure, making HDBSCAN report a high number of unclustered cells (the plotted version is provided by SAM's clustering procedure which has a postprocessing phase assigning all noisy cells to the closest cluster). Also notice that scVI tends to split clusters in two even if the reference clustering does not indicate this is the case.

G Clustering metrics

G.1 Adjusted rand index

The adjusted rand index (ARI) is a measure of the similarity between two clusters X and Y that is corrected for chance, mathematically defined as:

$$\frac{\sum_{ij}\binom{n_{ij}}{2} - \frac{\sum_i \binom{a_i}{2} \sum_j \binom{b_j}{2}}{\binom{n}{2}}}{\frac{1}{2}\left[\sum_i \binom{a_i}{2} + \sum_j \binom{b_j}{2}\right] - \frac{\sum_i \binom{a_i}{2} \sum_j \binom{b_j}{2}}{\binom{n}{2}}} \tag{12}$$

where n is the number of cells, and n_{ij}, a_i, and b_j are elements from a contingency table that summarises the overlap between X and Y [38]. X_i and Y_j denote groupings or partitions that are part of X and Y, respectively, i.e. $X = \{X_1, X_2, ...\}$ and $Y = \{Y_1, Y_2, ...\}$. n_{ij} represents the number of cells simultaneously assigned to X_i and Y_j. a_i and b_i are the sums of the ith row and jth column, respectively, in the contingency table.

G.2 Silhouette coefficient

The standard definition of the silhouette coefficient (SC) is given as the mean SC over all samples. The SC for a sample is computed using the mean intra-cluster distance (a) and mean nearest-cluster

distance (b) for that specific sample, using the following definition:

$$\text{SC}(i) = \frac{b_i - a_i}{\max(a_i, b_i)} \tag{13}$$

for a sample i. For b, the nearest cluster is the closest such that the sample is not part of it.

H Evaluation of different convolutions and values of k

We evaluated CellVGAE under different hyperparameter settings, including alternative values of k for the KNN/PKNN graphs and two additional graph convolutions. The experimental design is the same as for Table 2, and the graphs are generated using exactly the same KHVG and d parameters (where applicable). The results are provided in Supplementary Table 4 and Supplementary Table 5. To simplify the interpretation of these additional results, we used a second round of HDBSCAN clustering to assign the cells initially labelled as noisy to new groups. After applying this step no new noisy cells were identified, as most of the unsuccessful clusters of the first round were caused by very small clusters or proximity to larger ones.

	**GAT**				**GATv2**				**GCN**			
	**k = 3**		**k = 10**		**k = 3**		**k = 10**		**k = 3**		**k = 10**	
	**ARI**	**SC**	**ARI**	**SC**	**ARI**	**SC**	**ARI**	**SC**	**ARI**	**SC**	**ARI**	**SC**
Darmanis	0.9147	0.8187	0.9392	0.7750	0.8980	0.7505	0.9231	0.7296	0.9180	0.7254	0.9477	0.6895
Wang	0.8841	0.4646	0.8652	0.7212	0.8901	0.7909	0.8652	0.7923	0.9346	0.7767	0.8884	0.5689
Baron1	0.9667	0.8009	0.9551	0.7184	0.9665	0.7988	0.9524	0.7606	0.9663	0.8036	0.9651	0.7734
Baron2	0.9671	0.7805	0.9581	0.7331	0.9682	0.7970	0.9544	0.7032	0.9556	0.7600	0.9597	0.7627
Baron3	0.9706	0.7642	0.9678	0.7250	0.9699	0.7841	0.9715	0.6559	0.9676	0.7586	0.9690	0.7812
Baron4	0.8858	0.6473	0.8871	0.4597	0.8414	0.5805	0.9089	0.6698	0.8751	0.6492	0.8958	0.7119
Loh	0.9680	0.8756	0.9570	0.8265	0.9202	0.8699	0.9530	0.8191	0.9632	0.8431	0.9378	0.7612
Seger	0.9239	0.6978	0.9557	0.6722	0.9442	0.6928	0.9673	0.6858	0.9675	0.7144	0.9741	0.7268
Muraro	0.9242	0.7727	0.9458	0.7016	0.9449	0.8094	0.9418	0.7174	0.9452	0.8226	0.9492	0.7435

Supplementary Table 4: CellVGAE results when using three different convolutions: GAT, GATv2 and GCN, with different k values for the KNN/PKNN graph: 3 and 10 (Table 2 used $k = 5$).

	GAT				GATv2				GCN			
	k = 3		k = 10		k = 3		k = 10		k = 3		k = 10	
	**ARI**	**SC**	**ARI**	**SC**	**ARI**	**SC**	**ARI**	**SC**	**ARI**	**SC**	**ARI**	**SC**
Darmanis	0.9003	0.7306	0.9448	0.7457	0.9147	0.7364	0.9392	0.7618	0.9182	0.7264	0.9402	0.7434
Wang	0.9346	0.6119	0.8664	0.7106	0.8673	0.8367	0.8664	0.7558	0.9453	0.5911	0.9401	0.6601
Baron1	0.9642	0.7913	0.9653	0.7140	0.9667	0.8043	0.9556	0.7583	0.9660	0.8174	0.9682	0.7209
Baron2	0.9614	0.7885	0.9420	0.7672	0.9149	0.8156	0.8919	0.7771	0.9555	0.7945	0.9495	0.7060
Baron3	0.9695	0.7629	0.9612	0.7723	0.9672	0.7731	0.9651	0.7561	0.9689	0.8027	0.9615	0.7924
Baron4	0.8945	0.6518	0.8853	0.6636	0.8703	0.6749	0.8764	0.6202	0.9180	0.6484	0.9039	0.6779
Loh	0.9500	0.8157	0.9439	0.8094	0.9541	0.8402	0.9521	0.8265	0.9489	0.8070	0.9380	0.7962
Seger	0.9490	0.7082	0.9664	0.6536	0.9633	0.7304	0.9665	0.6722	0.9648	0.7187	0.9730	0.6772
Muraro	0.9451	0.7688	0.9442	0.7536	0.9464	0.7865	0.9384	0.7284	0.9503	0.8292	0.9260	0.7365

Supplementary Table 5: CellVGAE results when using three different convolutions: GAT, GATv2 and GCN, with different k values for the KNN/PKNN graph: 3 and 10 (Table 2 used k = 5) and a decoder neural network that reconstructs the gene expression values.

I Datasets

We collect and label the datasets used throughout this work, along with the accompanying publication, size and source of download in Supplementary Table 6. The number of cells is reported after quality control (for RETINA this means running the original R clean-up code provided by the authors: https://github.com/broadinstitute/BipolarCell2016).

As described in *Results*, we decided to use the same 9 datasets utilised in [12]. The one exception is the dataset *Loh* – where SAM results are reported on a version of the dataset with 651 cells; however, while we were able to find this version on the conquer[3] database, it contains over $65{,}000$ rows (genes) and we have discovered that the Seurat code used for selecting the top HVGs (see below) does not work well for this amount of information, i.e. it leads to untrainable neural models with very poor performance (both CellVGAE and DiffVAE). For this reason, we have replaced the dataset with a filtered version of 498 cells and $20{,}142$ rows, provided by the original publication authors, amenable to HVG selection, but we acknowledge that SAM works successfully even on the 651 cells version. We also note that the DiffVAE authors have already benchmarked the method on a different version of the *Muraro* dataset, with $2{,}285$ cells, hence the results are not comparable. We were unable to find this version online, and since SAM uses the version with $2{,}126$ cells, we use it as well, for CellVGAE, DiffVAE and scVI.

J Experimental design

As we aim to fairly and comprehensively evaluate all the methods, we now describe our experimental design.

J.1 Dataset preprocessing

We assume the data has already passed quality control (except for the *Wang* dataset, which comes with 178 low-quality samples that are discarded by us and by SAM). For SAM, we input the count matrix as is, since the algorithm has its own preprocessing method and works on the entire gene expression matrix. For CellVGAE and DiffVAE, we log-normalise the data and select the most variable genes using Seurat (`NormalizeData()` and `FindVariableFeatures()` functions). scVI uses the raw count matrices as input. For all scVI experiments in Table 2 we select the top $1{,}200$

[3] http://imlspenticton.uzh.ch:3838/conquer/

Name	Publication	# of cells	Source
S. mansoni	[12]	338	SAM official repository
PBMC3k		2,638	10X Genomics
Darmanis	[39]	420	single-cell-sota
Wang	[40]	457	hemberg-lab
Baron1	[41]	1,937	Gene Expression Omnibus, supplementary file
Baron2	[41]	1,724	Gene Expression Omnibus, supplementary file
Baron3	[41]	3,605	Gene Expression Omnibus, supplementary file
Baron4	[41]	1,303	Gene Expression Omnibus, supplementary file
Loh	[42]	498	Cell, supplementary file
Segerstolpe	[43]	2,209	hemberg-lab
Muraro	[44]	2,126	hemberg-lab
RETINA	[33]	26,439	scVI-reproducibility repository

Supplementary Table 6: Summary of the used datasets.

HVGs as used in the official demo code and follow all the remaining steps ($1{,}000$ HVGs are used in the training time benchmark).

For the 9 datasets we matched the number of HVGs for DiffVAE (same as CellVGAE) for a fair comparison. scVI uses its default of $1{,}200$ genes for all datasets as even these are underperforming compared to DiffVAE, thus it makes sense to show the best that it is capable of. For RETINA we used $1{,}000$ HVGs for CellVGAE and $1{,}200$ for DiffVAE and scVI.

The Seurat representations were achieved using the functions `RunPCA()` and `RunUMAP()` with 10 dimensions. For *Schistosoma mansoni* we run SAM with default settings (as the documentation advises for this particular dataset). Whenever we run SAM for other datasets, we perform a hyperparameter search with parameters $\texttt{k} \in \{5, 10, 20, 40, 80, 120, 160, 200\}$ (number of nearest neighbours) and $\texttt{num\_norm\_avg} \in \{1, 5, 10, 25, 50, 100, 150, 200, 800\}$ (number of largest spatial dispersions to average), all values being provided in the official documentation, for a total of 72 configurations. Only the best performing models of SAM according to this search are shown in this study. We do not illustrate other methods as SAM already includes an exhaustive evaluation against the most widely used tools at the time.

J.2 Graph generation

We generally build the KNN graphs from the selected HVGs with the scran function `buildKNNGraph()`. Slight variations are possible and were found to increase performance, for example in the form of applying d-dimensional PCA before the nearest neighbour search. This is applied by specifying a `d` parameter to the `buildKNNGraph()` function. For the *Darmanis* dataset, building the KNN graph from the entire log-normalised gene expression matrix, while also applying PCA helped (the node features are still the 250 HVGs). Alternatively, for 2 out of the 9 datasets and for RETINA we use the PKNN (each cell is connected to k other cells with the highest correlation values). Detailed information about the setup used for each dataset is provided in the repository. For RETINA, building KNNs using `scran` is slow; instead, we successfully experimented with Faiss, which can compute the exact KNN graph for this dataset in seconds on a modern multi-core processor.

J.3 Architecture

Architecturally, by default CellVGAE is configured with a two-layer encoder with 128 hidden dimensions, 50 latent dimensions and the same settings of learning rate and batch size for all of the 9 datasets, only lowering the number of attention heads for the larger datasets. All the experiments corresponding to the 9 datasets are performed on a consumer-grade mid-range GPU equipped with 8GB of VRAM (NVIDIA RTX 2070) and we did not hit any memory limitations when using the KL loss. A large number of attention heads is not necessarily required for top performance, but we increased this hyperparameter to stabilise learning, following the advice of the GAT authors. The default PyTorch Geometric implementation of the KL loss is considerably more memory efficient than the MMD loss and we recommend it for large datasets. DiffVAE parameters are based on the default, recommended settings and generally mirror CellVGAE's, with two layers of sizes $\{256, 128\}$ in the encoder and decoder, and 50 latent dimensions. For the RETINA dataset, we used two layers with 256 hidden dimensions for all deep learning architectures, 50 latent dimensions and otherwise the default settings for each architecture.

J.4 Experimental pipeline and training

We observed that running SAM with default parameters can lead to poor performance on several datasets. This is not unexpected as the datasets vary greatly in size, composition and scRNA-seq protocols. To ensure we show the best that the method is capable of, we run SAM with

$\texttt{k} \in \{5, 10, 20, 40, 80, 120, 160, 200\}$ and $\texttt{num\_norm\_avg} \in \{1, 5, 10, 25, 50, 100, 150, 200, 800\}$, as previously described, and report only the best value.

For the neural approaches, a complication that arises is that the ARI cannot be optimised directly. To combat this, we first use a 90/10 split for each dataset and monitor the validation loss, to detect the point of convergence and then (eventually) overfitting. Once this is established, we propose a universal strategy where we train different models for 100, 120, 140, 160, 180, 200 and 250 epochs for DiffVAE, and 200, 220, 240, 260, 280, 300 and 400 epochs for CellVGAE, on the whole dataset. We run each configuration 20 times, for a total of 140 trials for each dataset, or 1,260 trials in total, for each of CellVGAE and DiffVAE. Since both methods rely on clustering applied to the two-dimensional UMAP projection of the latent embeddings, the UMAP representation itself is important. UMAP is not deterministic unless using the same seed and we have observed small variations in the resulting ARI scores depending on the parameters of UMAP. To filter out irrelevant representations, we run UMAP 30 times with random initialisation, for each trial. Finally, for each resulting projection, we run HDBSCAN 21 times with the `min_cluster_size` and `min_samples` parameters locked to the same value in $\{10, 11, ..., 30\}$. Due to the large variability in data quantity and structure, a single clustering setting would be unable to perform well across all datasets and projections. As such, we remove any bias by using a large search space. After applying all the steps above, we select the best model in terms of ARI and report it, along with the silhouette coefficient, the number of found clusters and noise, i.e. cells classified as '-1' by HDBSCAN (Table 2).

scVI is used with default settings, adjusted to always use two hidden layers of size 256 and a latent size of 50. Since scVI uses a warm-up procedure under the hood we do not perform trials with different numbers of epochs. Instead, for each of the 7 datasets (2 out of 9 are not available as they do not provide the raw counts) we run 40 trials using the same settings and report the best version according to the internal clustering procedure.

All the previous details apply to the 9 datasets. For RETINA, all deep learning methods use two hidden layers of size 256, a latent dimension of 50, and are trained for 400 epochs. 40 trials are run for each method and the best is reported. The parameter search-space for SAM is the same.

J.5 Experimental platform

CellVGAE was developed using PyTorch [46] and PyTorch Geometric [47], at version 1.6.0 and 1.6.1, respectively (also tested successfully with versions 1.7.0, 1.8.1, and 1.9.0 of PyTorch). The

other tools and libraries used the most recent version available for Python 3.9 as of the second half of 2021. The plots use matplotlib [48], seaborn [49] or Plotly [50]. Seurat 3 was installed on R version 4.0.2 [51]. The main development operating system was Windows 10, running the latest insider developer build available at the time. Tools available exclusively on Linux were installed and used on Ubuntu 20.10 running on the same hardware. All CellVGAE models for the 9 datasets were trained on an NVIDIA GeForce RTX 2070 GPU with 8GB VRAM connected through Thunderbolt 3 to a machine equipped with an Intel Core i9-8950HK and 32GB of DDR4 memory. The used CUDA toolkit version is 10.2 for PyTorch 1.6.0, 11.0 for PyTorch 1.7.0 and 11.1 for PyTorch 1.9.0. For the 9 datasets, DiffVAE was run exclusively on the CPU, using a compatible version of TensorFlow (1.15).

All experiments involving RETINA or timing and memory benchmarks were carried out on a platform using an NVIDIA GeForce RTX 3090 GPU with 24GB of video memory, an AMD Ryzen 5950X processor and 32GB of DDR4 memory, using PyTorch 1.8.1, PyTorch Geometric 1.7.0 and CUDA 11.1. For DiffVAE experiments on RETINA we used TensorFlow 2.4 GPU, where we replaced all imports to use `TensorFlow.compat.v1`.

All timing and memory experiments from Figure 8 used the same NVIDIA GeForce RTX 3090 platform or a cluster equipped with an NVIDIA Tesla V100 with 32GB of video memory.

SAM version 0.7.6 was used throughout the paper.

K Discovered number of clusters

For Table 2, if some cells are excluded due to HDBSCAN parameters (e.g. minimum cluster size too high for the very small clusters) but would otherwise form a well-defined cluster, they are not counted towards noise and contribute towards the # metric in Table 2. Thus, the cells included as noise are only those who are too close to existing clusters to be properly classified.

None of the methods is consistent in finding the true number of clusters, but arguably SAM is the closest. However, since this is not reflected in an increase in SAM's ARI, we cannot say that it is meaningful. On the other hand, many clusters are extremely small, such as *Baron4* with 1 epsilon cell, 1 mast, 1 Schwann, 1 T, 2 acinar, 5 quiescent stellate and 7 endothelial cells. Realistically, we would not expect most methods, especially deep learning, to detect these clusters, although CellVGAE manages to find and separate the 10 macrophage cells (extreme left in the leftmost plot in Supplementary Figure 7f).

Dataset	**Truth**	**SAM**	**CellVGAE**	**DiffVAE**	**scVI**
Darmanis	8	8	8	7	5
Wang	7	7	5	5	N/A
Baron1	14	11	11	10	9
Baron2	14	10	9	8	9
Baron3	14	8	10	8	7
Baron4	14	8	7	7	8
Loh	9	9	9	9	N/A
Segerstolpe	14	15	10	9	8
Muraro	10	9	8	8	9
RETINA	15 (16)	14	16	15	14

Supplementary Table 7: Comparison of the number of found clusters on the 10 datasets for all evaluated methods. For RETINA, the authors suggest a possible subclustering of one of the smaller clusters, thus either 15 or 16 is a valid answer.

L Hyperparameters and CellVGAE graph settings for the 1.3 million cells dataset

**Subset size**	**# edges**	**Cutoff**
25K	17,801	$-0.1 \times$ std
50K	46,814	$-0.1 \times$ std
100K	142,059	$-0.01 \times$ std
150K	165,387	$-0.1 \times$ std
300K	301,712	$-0.1 \times$ std
500K	310,689	$-0.1 \times$ std
1M	525,777	$-0.25 \times$ std
Full	624,869	$-0.275 \times$ std

Supplementary Table 8: The number of edges and cutoffs used for the eight subsets of the 1.3 million mouse brain cells datasets.

KNN graphs corresponding to each subset were generated using Faiss, with $k = 3$. As the number of edges can grow extremely quickly for large datasets, we implement a cutoff based on the distances computed by Faiss (all discussed KNN graphs are based on the Euclidean distance, but other metrics are available). More specifically, the interface allows adding or subtracting a multiple of the standard deviation of the distances to the mean value. To reduce the number of edges a negative cutoff value

is used, restricting it to smaller distance values (closer cells). A cutoff value of -0.1 was selected as the default choice, except for the *100K* dataset which resulted in a very small number of edges. Thus, a value of -0.01 was used in this particular case.

Changes for *1M* and *Full*

For the two largest subsets (*1M* and *Full*), more restrictive cutoff values were used: -0.25 and -0.275, respectively, for the RTX 3090 models and -0.25 for both subsets when using the V100 platform. This step is designed to eliminate potential spurious edges for over 1 million cells and to allow the CellVGAE model to fit in 24GB of video memory.

Furthermore, for the RTX 3090 models, the hidden dimensions of the first two layers were adjusted to a size of 64 each, and for the *Full* model the number of attention heads for the $\boldsymbol{\mu}$ and $\boldsymbol{\sigma}$ layers was reduced to 1. The *Full* V100 models used the same adjusted number of attention heads but used 100 and 64 as the hidden size for the first two layers (default was 128 and 64).

Hyperparameters

CellVGAE used KNN graphs with k $= 3$ generated by Faiss for each subset consisting of the top $1{,}000$ HVGs, two hidden layers of dimensions 128 and 64 (size 64 for the $\boldsymbol{\mu}$ and $\boldsymbol{\sigma}$ layers), 50 latent dimensions and 3 attention heads per layer. DiffVAE and scVI equivalently used the same gene expression information and hyperparameters, except the graph information. As scVI does not allow two different layer sizes, we used two layers with a dimension of 117, which is the closest to the number of connections in a dense network with 128 and 64 neurons per layer. All neural models were trained for 200 epochs.

M CellVGAE reconstructed graphs

In this section, we visualise the graphs reconstructed by CellVGAE as adjacency matrices, for values of k in the set $\{5, 10, 15, 20, 25, 30, 35, 40, 45, 50, 100\}$ on the *Schistosoma mansoni* dataset (338 cells), where the CellVGAE model was trained with 500 HVGs and KHVG=100. We plot the original adjacency matrix in the top rows, the reconstructed (continuous) adjacency matrix in the middle rows, and the overlap matrix between the binarised reconstructed matrix and the original in the bottom rows, where the overlap matrix has ones in positions where both matrices are one,

otherwise zeros. The binarisation transforms continuous values greater than or equal to 0.5 to ones, otherwise to zeros. The plots are provided in Supplementary Figures 8 to 11.

The plots indicate that CellVGAE reconstructs sparser matrices preserving the same patterns present in the original adjacency matrices.

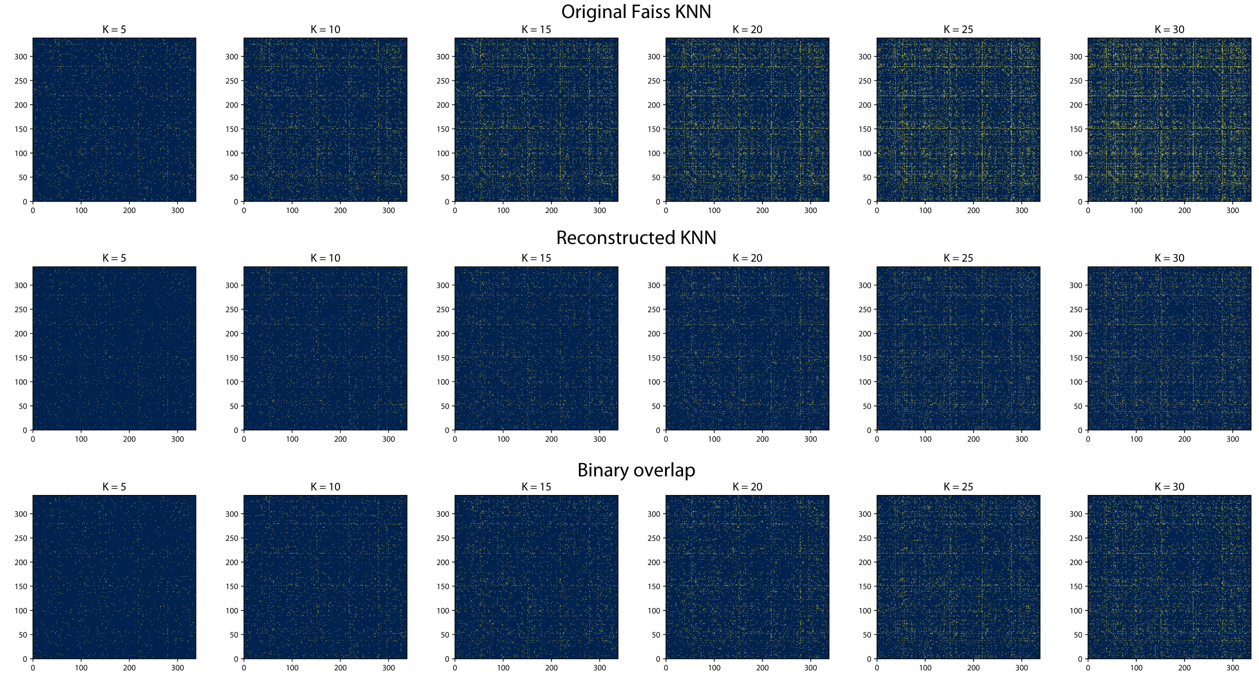

Supplementary Figure 8: Original, reconstructed and binary overlap adjacency matrices for Faiss KNN graphs with k from 5 to 30.

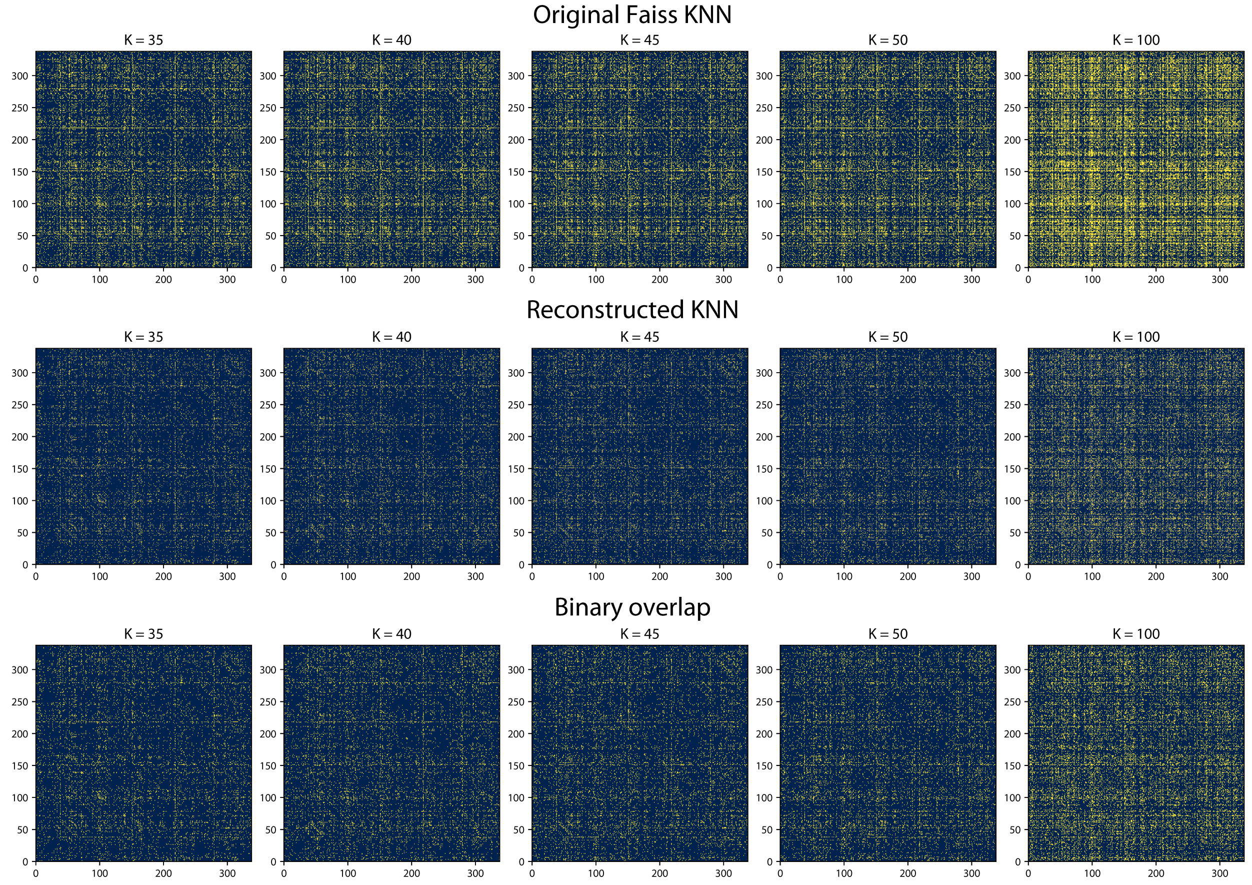

Supplementary Figure 9: Original, reconstructed and binary overlap adjacency matrices for Faiss KNN graphs with k from 35 to 100.

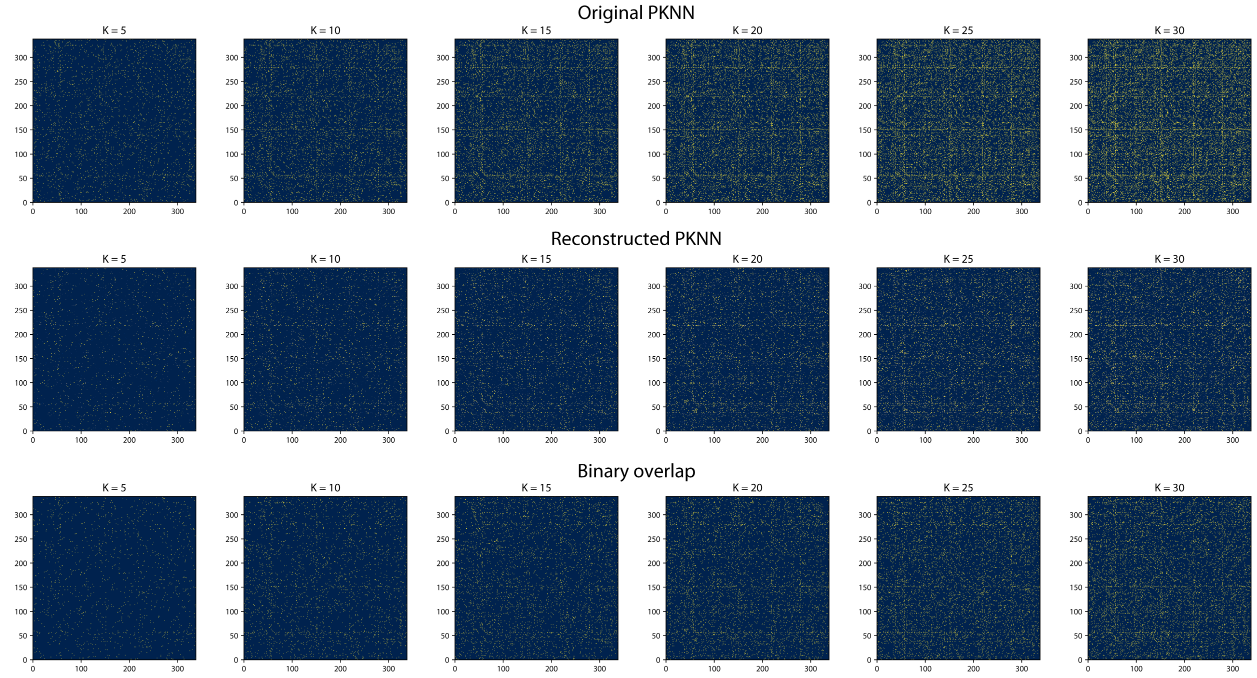

Supplementary Figure 10: Original, reconstructed and binary overlap adjacency matrices for PKNN graphs with k from 5 to 30.

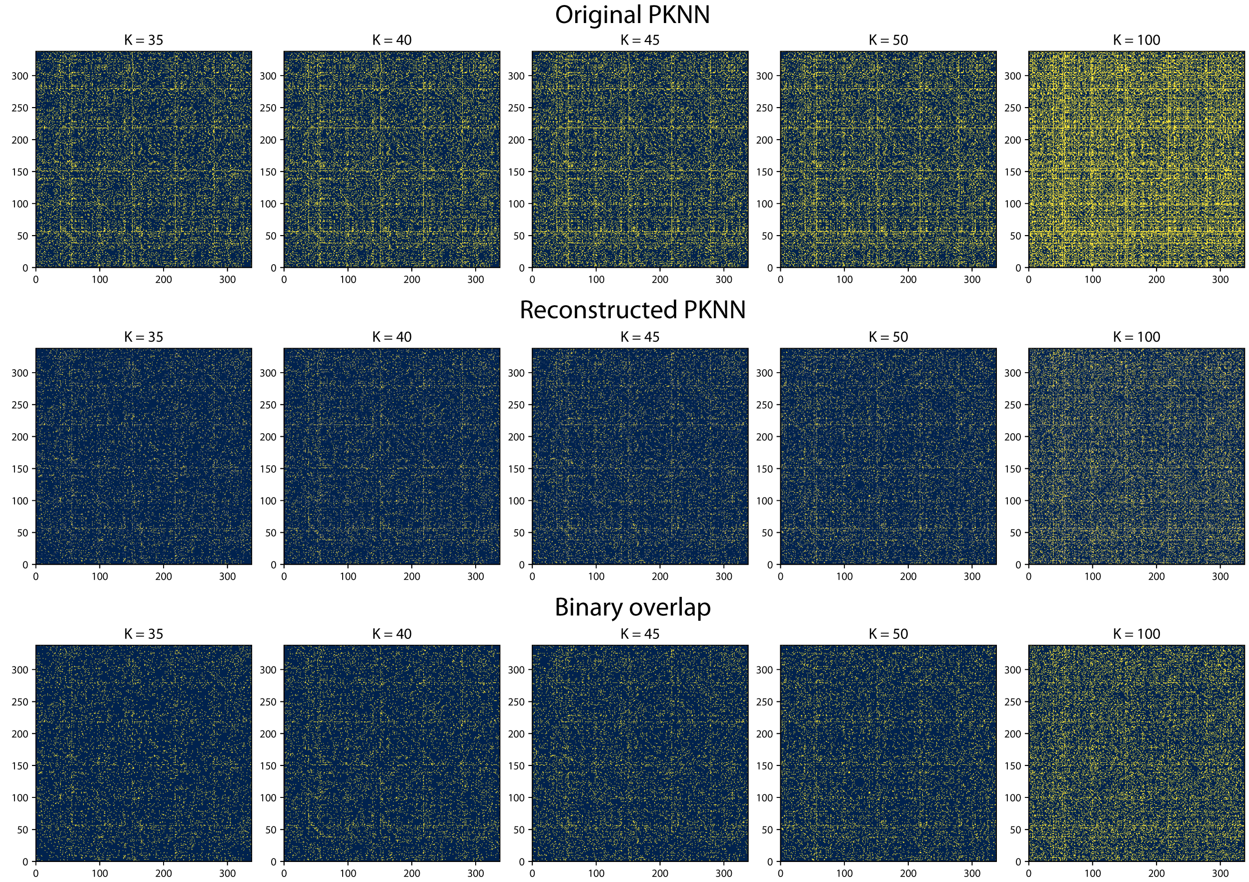

Supplementary Figure 11: Original, reconstructed and binary overlap adjacency matrices for PKNN graphs with k from 35 to 100.

N Visualisation of all the attention heads

In this section, we apply the same visualisation techniques of Figures 6a and 6b to the individual attention heads, for each layer of the two models. To better understand the differences between the attention heads, we subtract each head's values from the mean across all heads, and apply a colour overlay only to the nodes passing a certain threshold, the others being illustrated only with a black outline. The code and settings for generating the figures is available in the repository.

N.1 PBMC3k

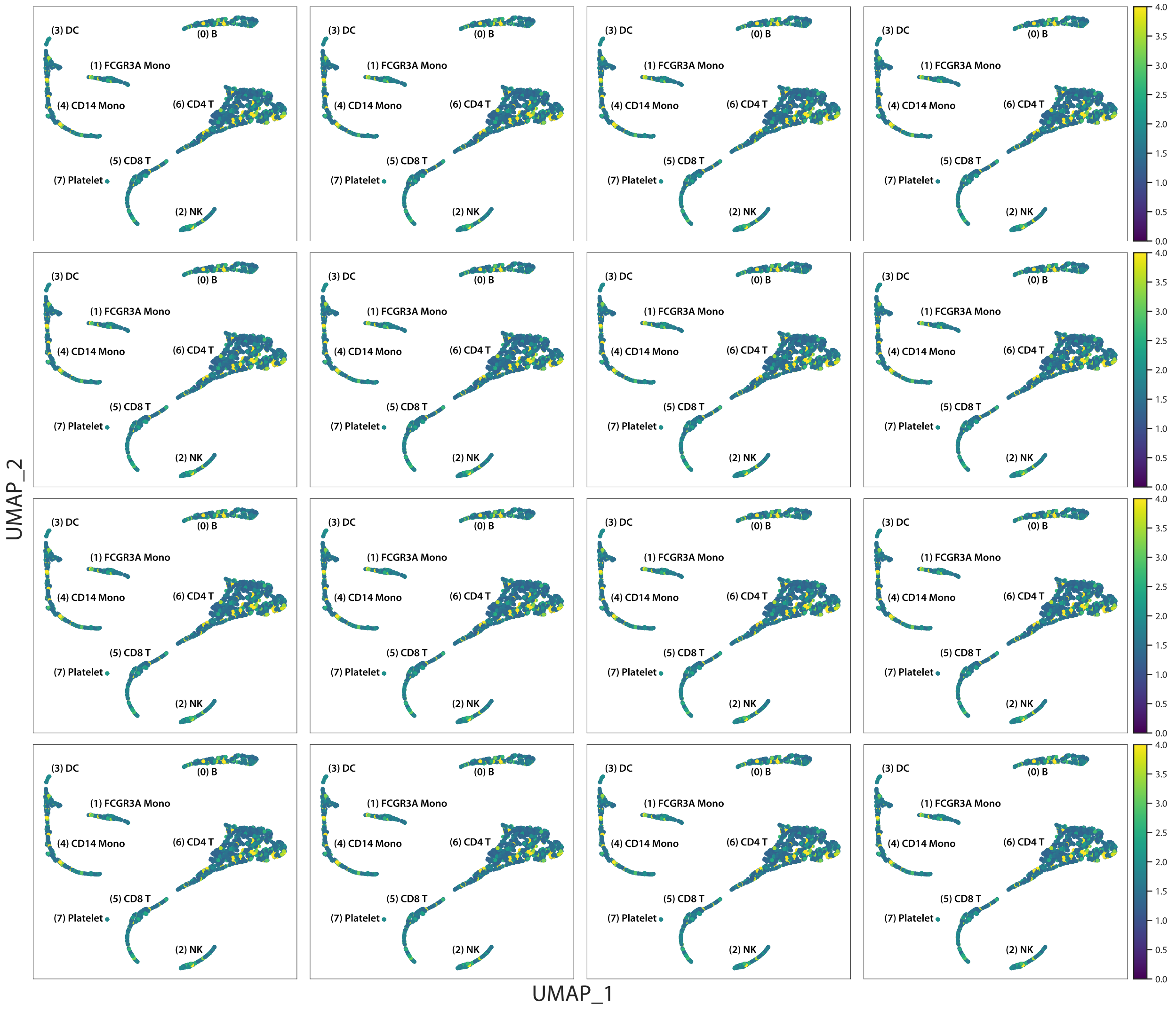

Supplementary Figure 12: All 16 attention heads of the *PBMC3k* CellVGAE model corresponding to the first layer.

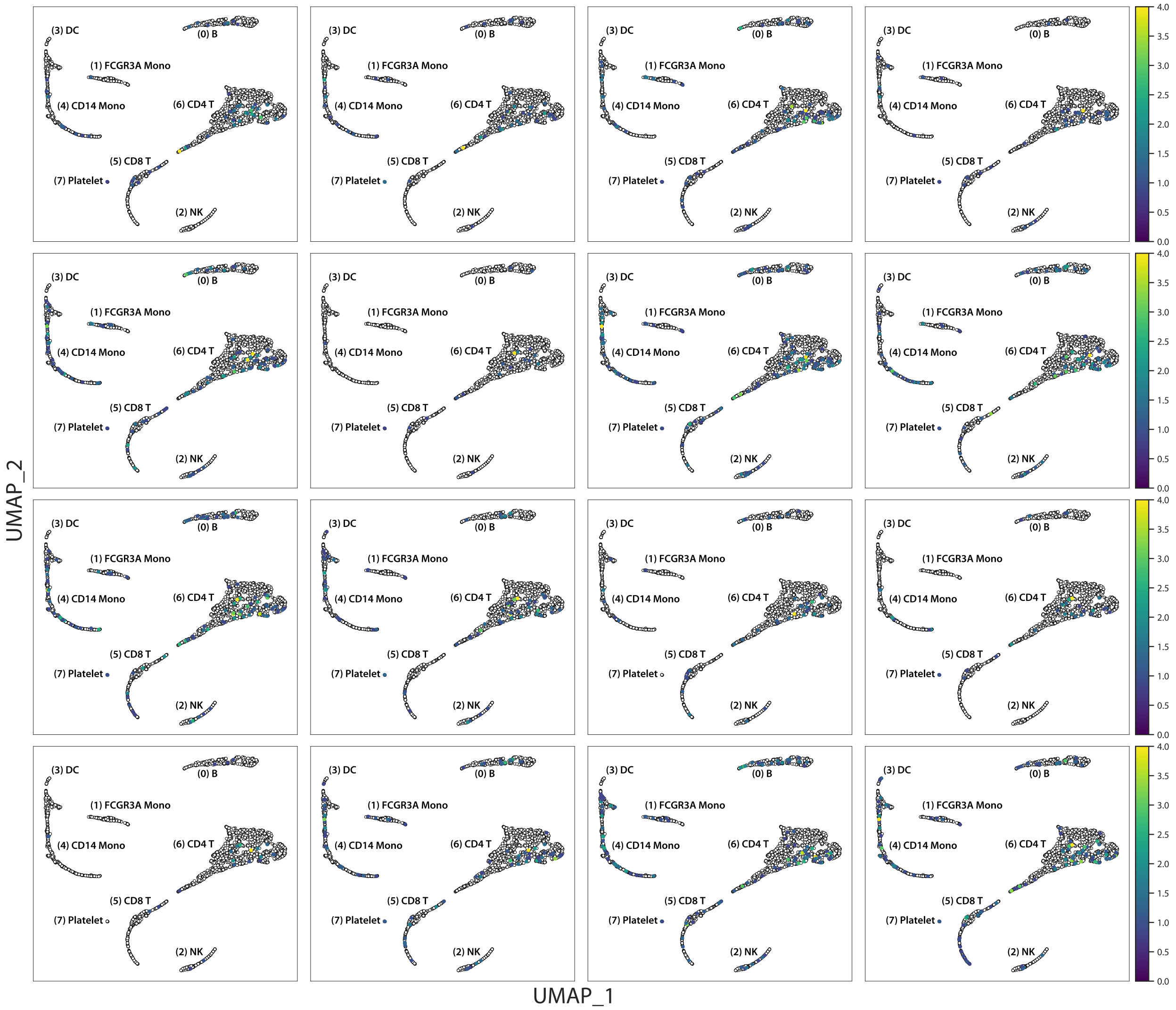

Supplementary Figure 13: Difference between each head's values and the mean, for all 16 attention heads of the *PBMC3k* CellVGAE model corresponding to the first layer.

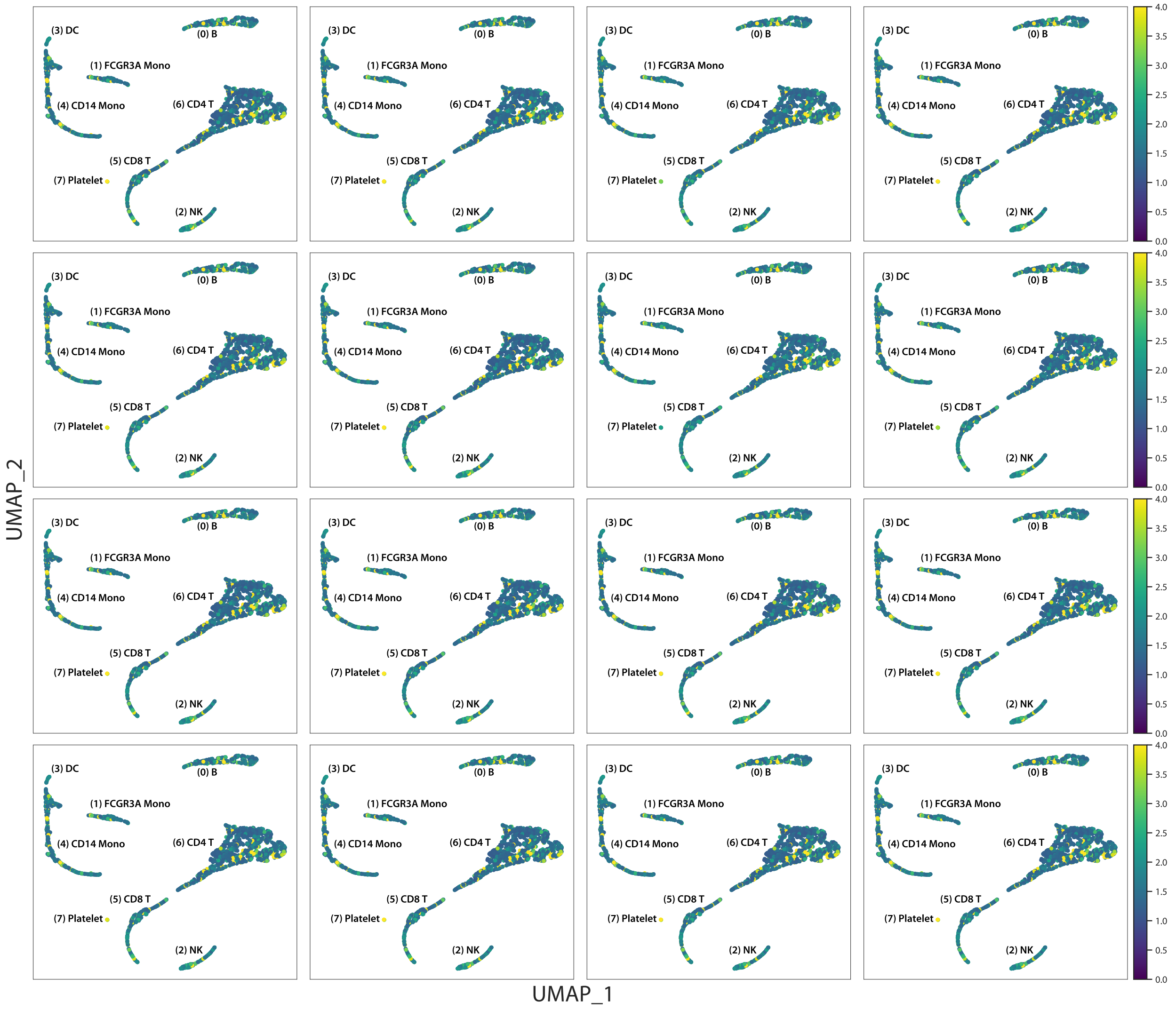

Supplementary Figure 14: All 16 attention heads of the *PBMC3k* CellVGAE model corresponding to the second layer.

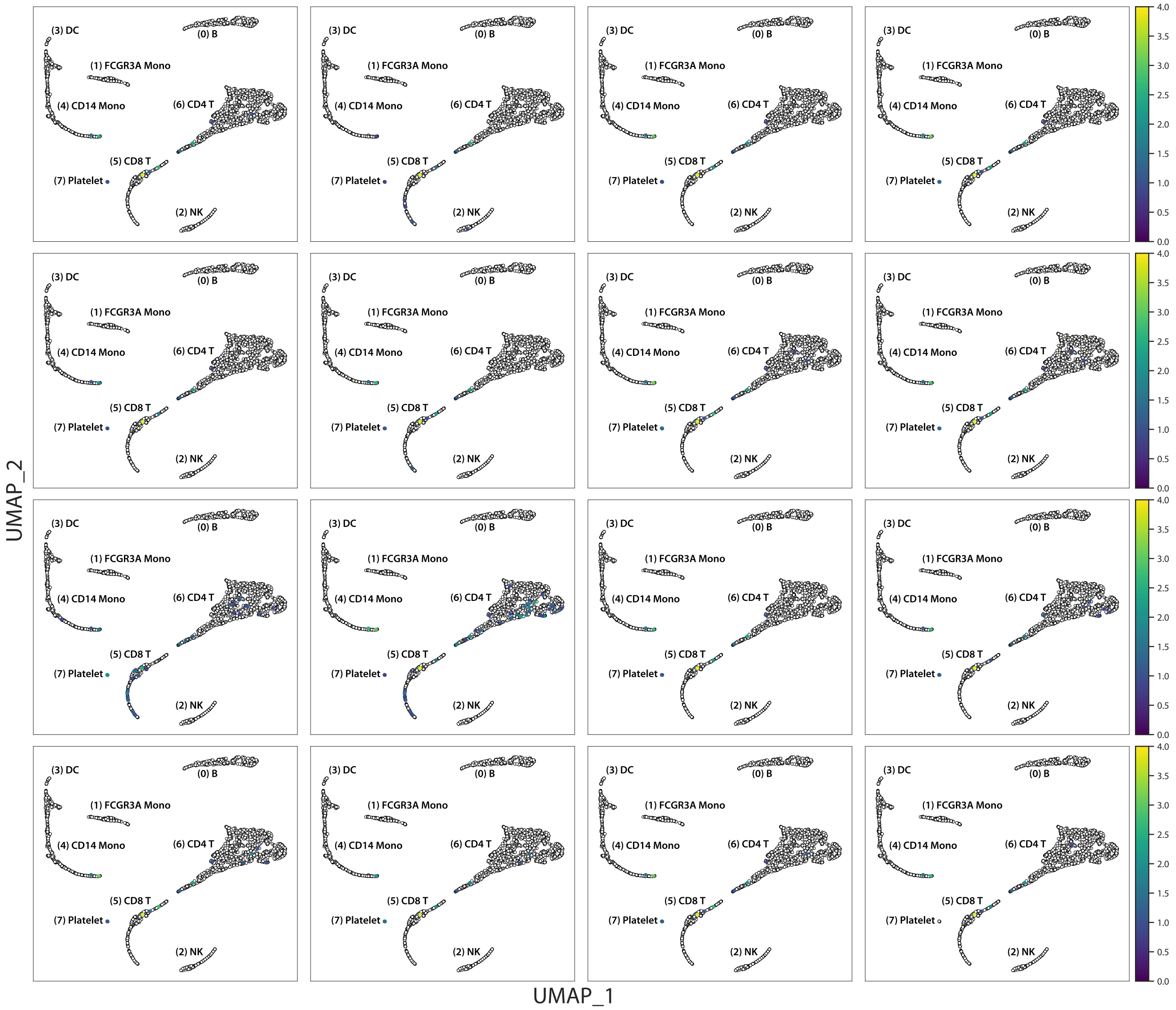

Supplementary Figure 15: Difference between each head's values and the mean, for all 16 attention heads of the *PBMC3k* CellVGAE model corresponding to the second layer.

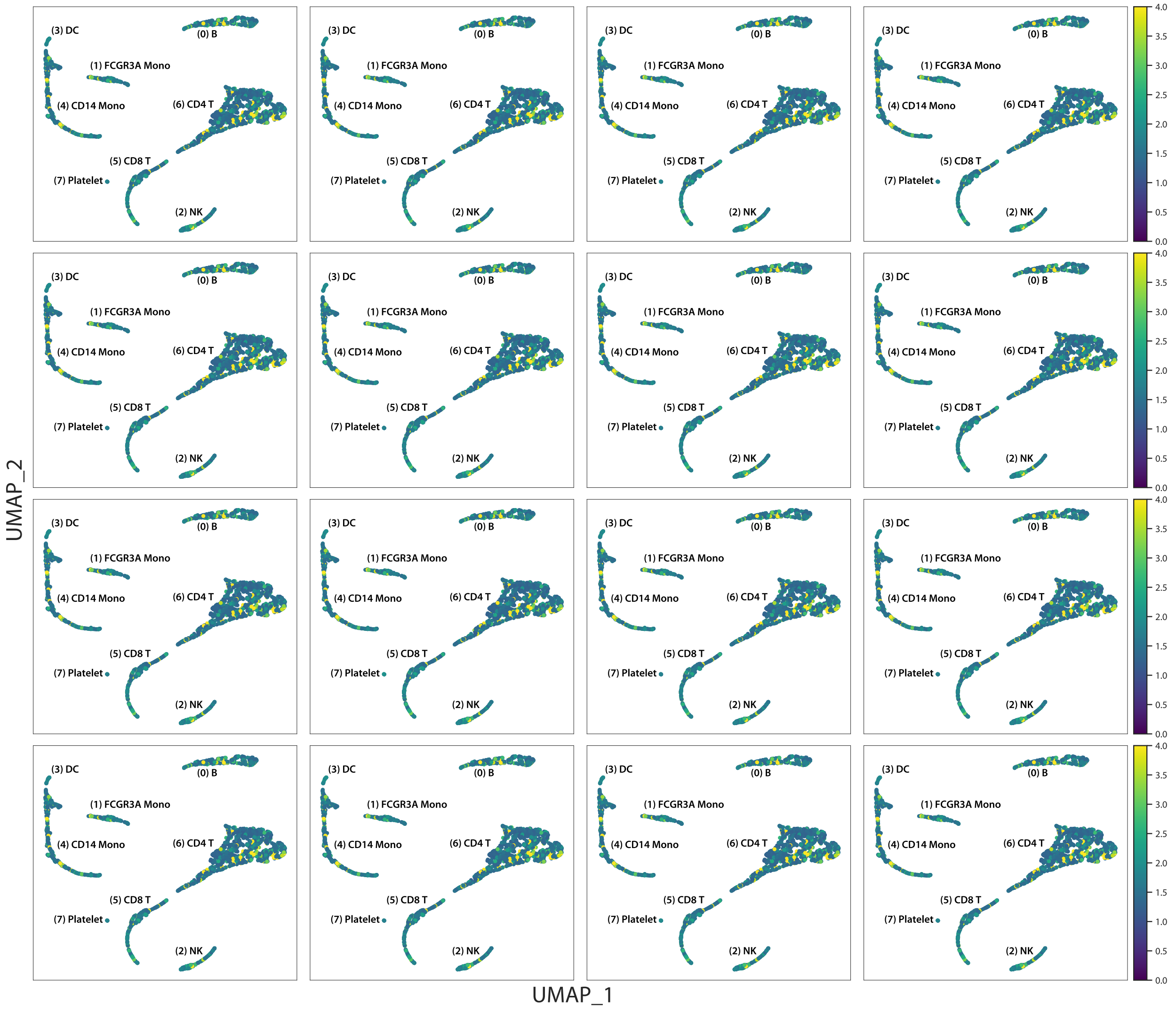

Supplementary Figure 16: All 16 attention heads of the *PBMC3k* CellVGAE model corresponding to the mean (μ) layer.

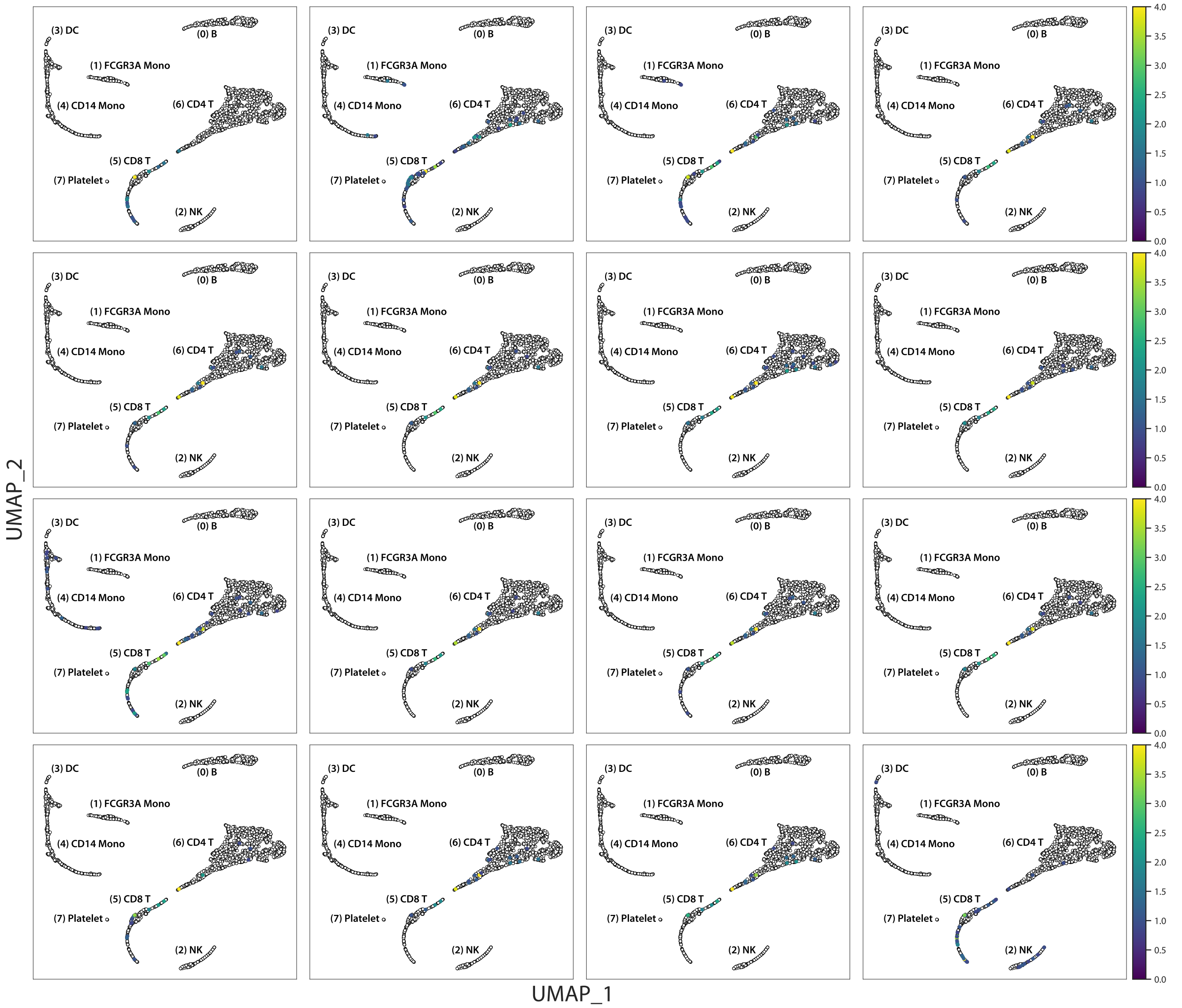

Supplementary Figure 17: Difference between each head's values and the mean, for all 16 attention heads of the *PBMC3k* CellVGAE model corresponding to the mean (μ) layer.

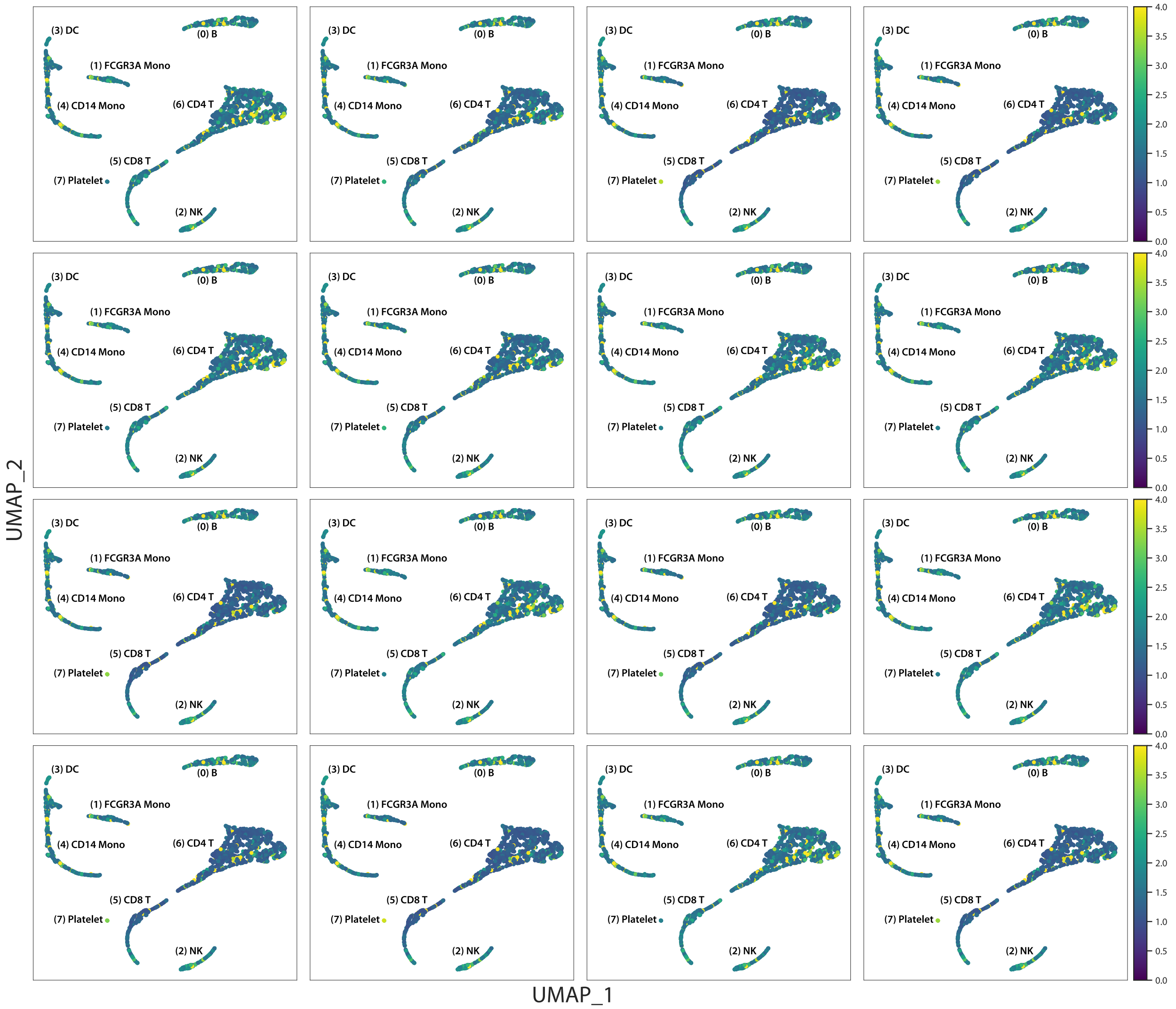

Supplementary Figure 18: All 16 attention heads of the *PBMC3k* CellVGAE model corresponding to the std (σ) layer.

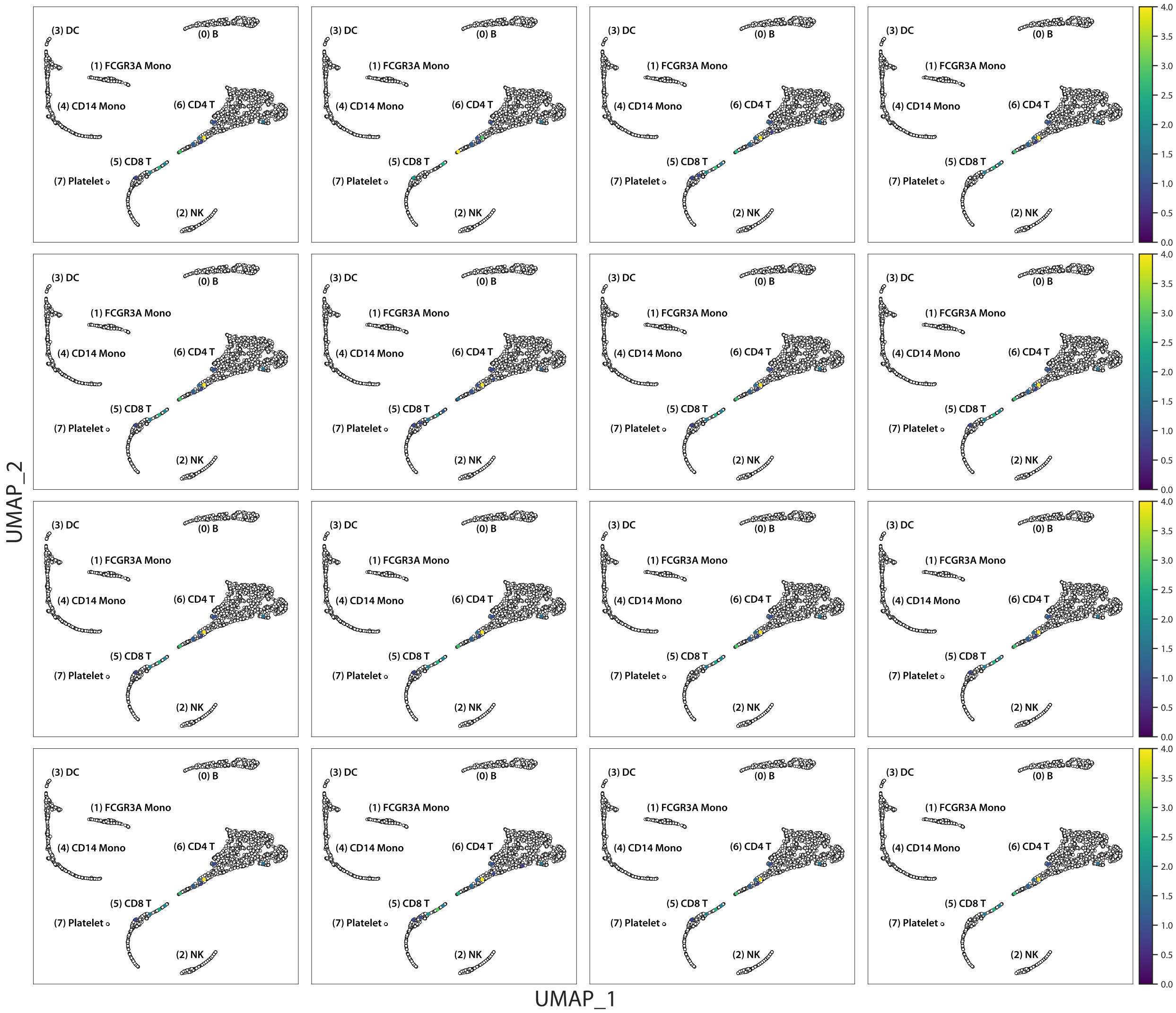

Supplementary Figure 19: Difference between each head's values and the mean, for all 16 attention heads of the *PBMC3k* CellVGAE model corresponding to the std (σ) layer.

N.2 Darmanis

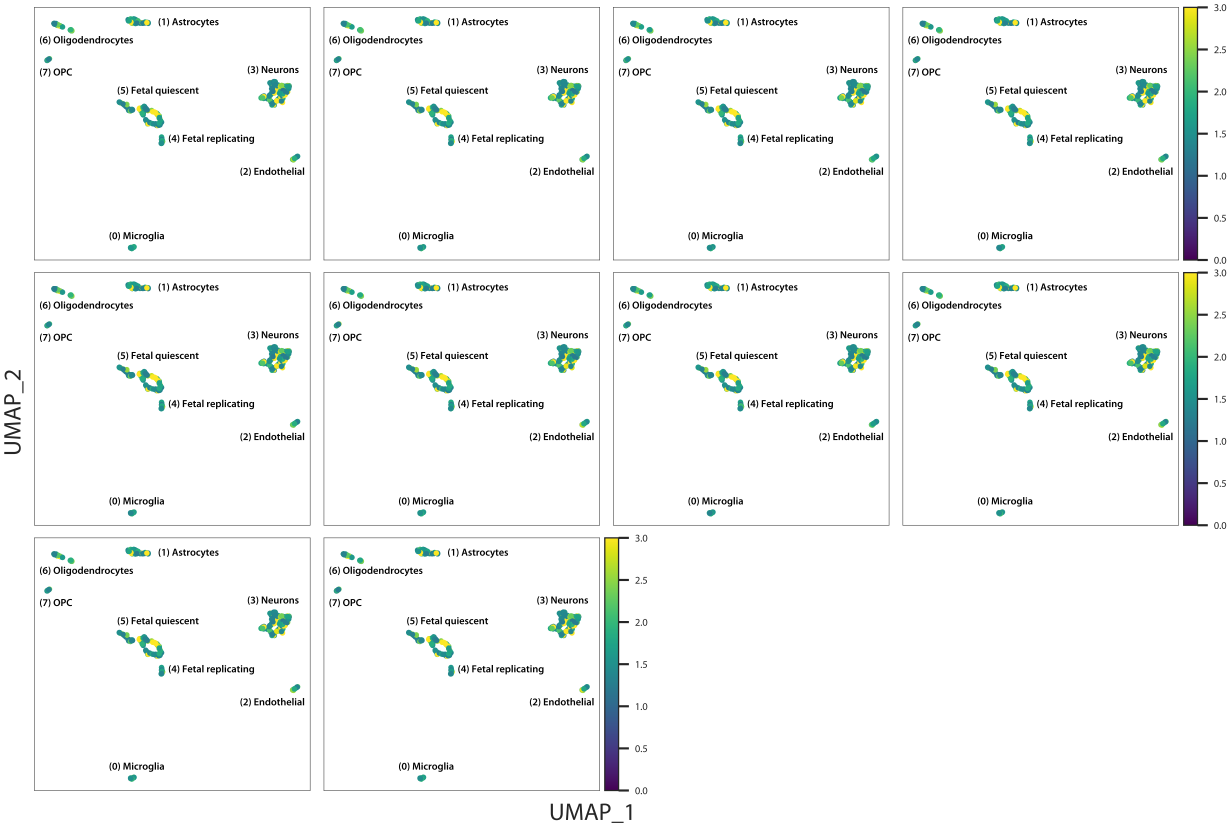

Supplementary Figure 20: All 16 attention heads of the Darmanis CellVGAE model corresponding to the first layer.

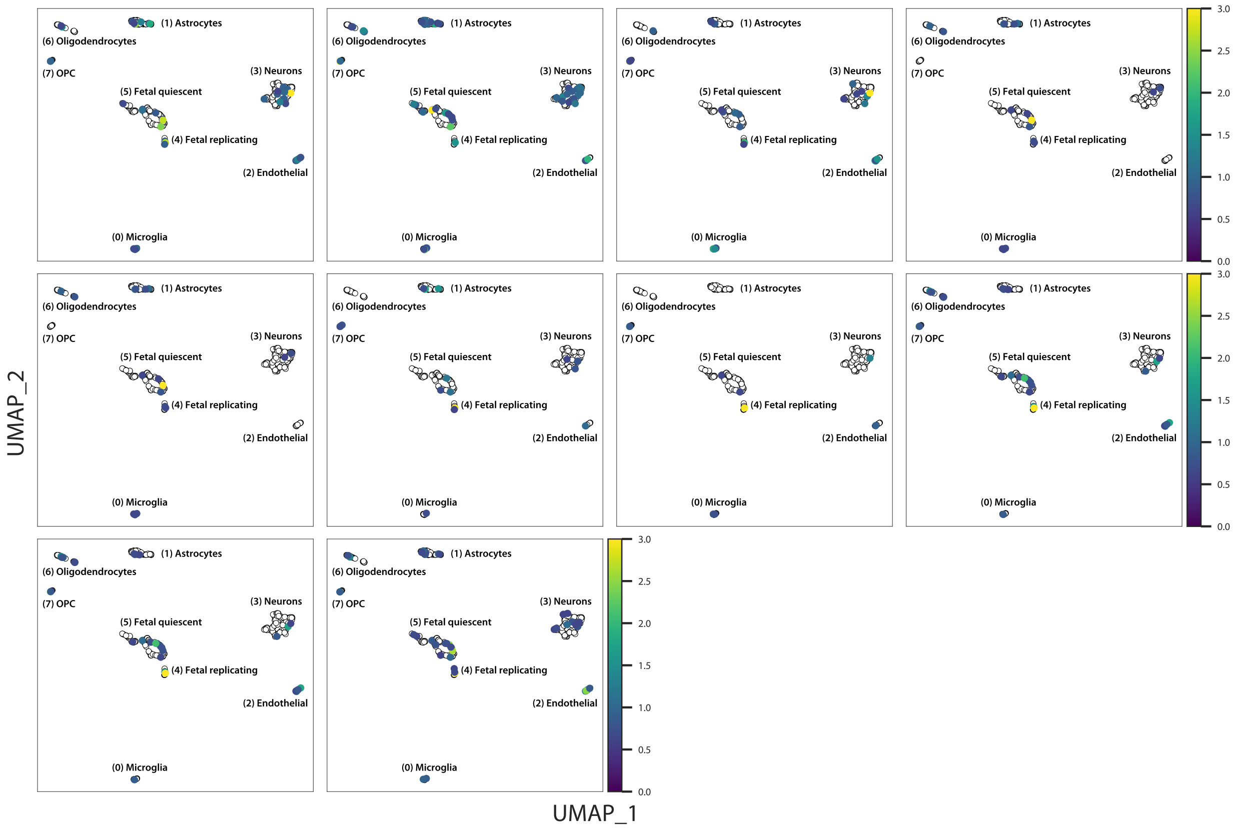

Supplementary Figure 21: Difference between each head's values and the mean, for all 16 attention heads of the Darmanis CellVGAE model corresponding to the first layer.

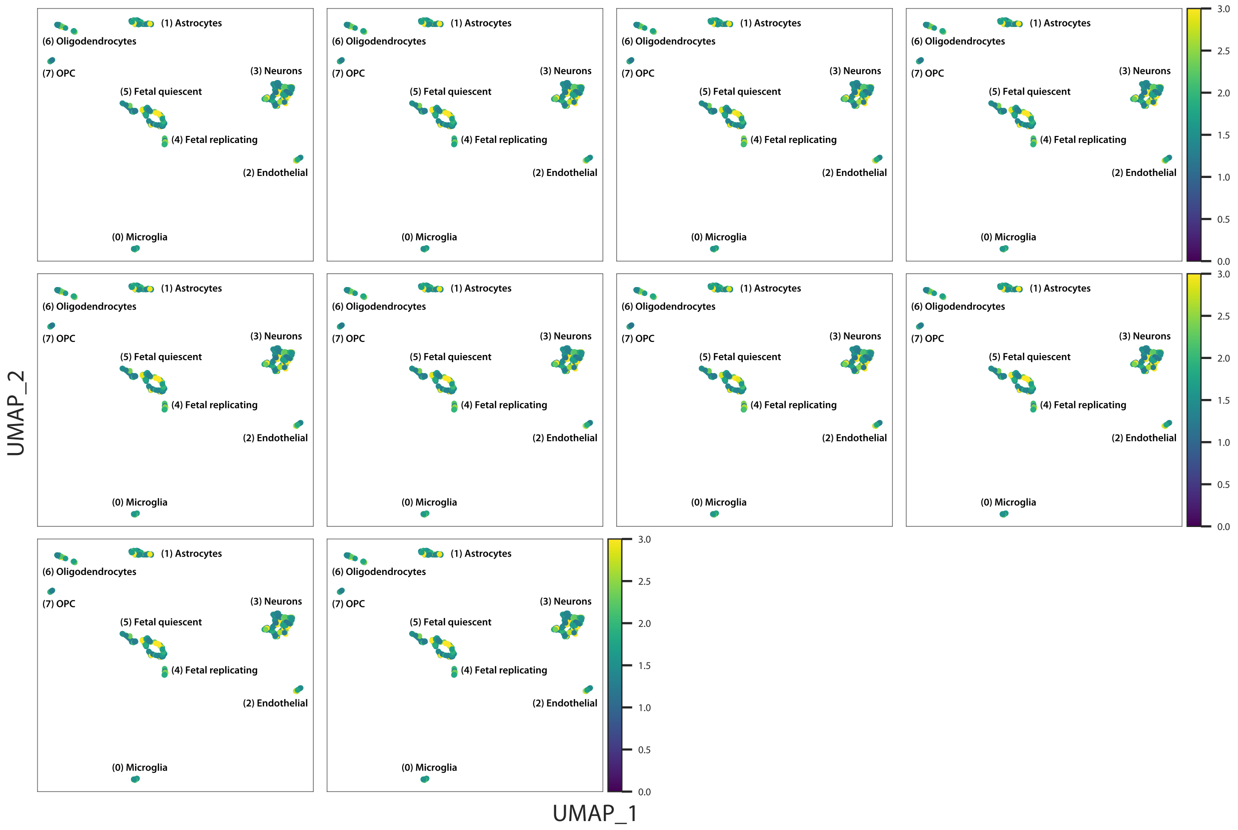

Supplementary Figure 22: All 16 attention heads of the Darmanis CellVGAE model corresponding to the second layer.

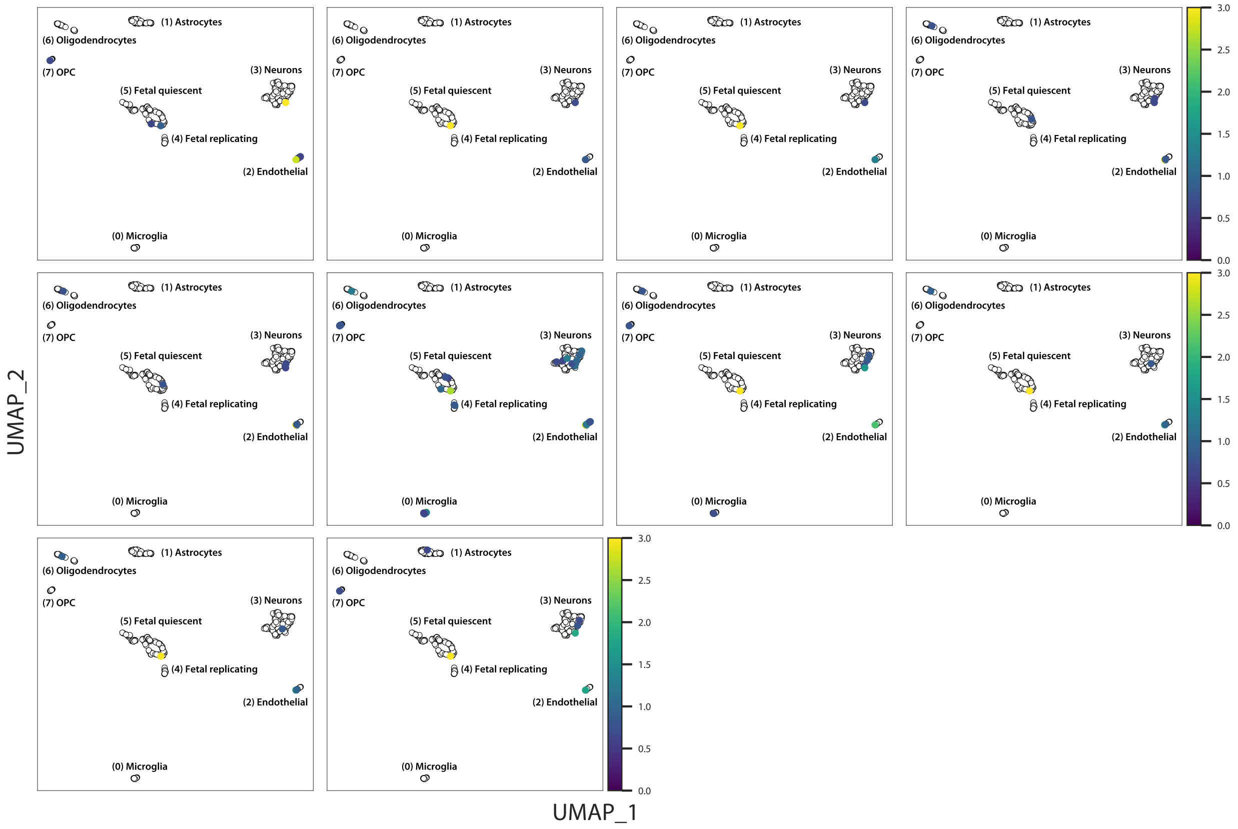

Supplementary Figure 23: Difference between each head's values and the mean, for all 16 attention heads of the Darmanis CellVGAE model corresponding to the second layer.

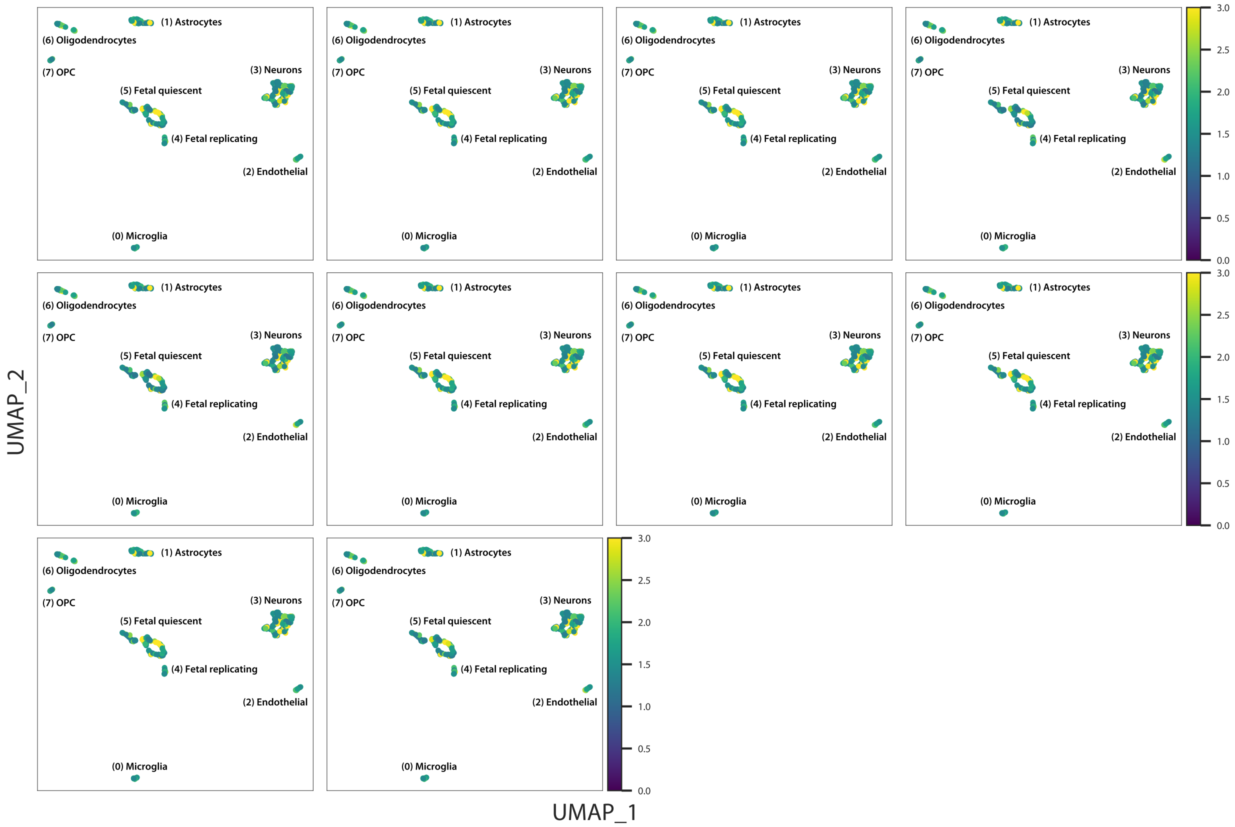

Supplementary Figure 24: All 16 attention heads of the Darmanis CellVGAE model corresponding to the mean (μ) layer.

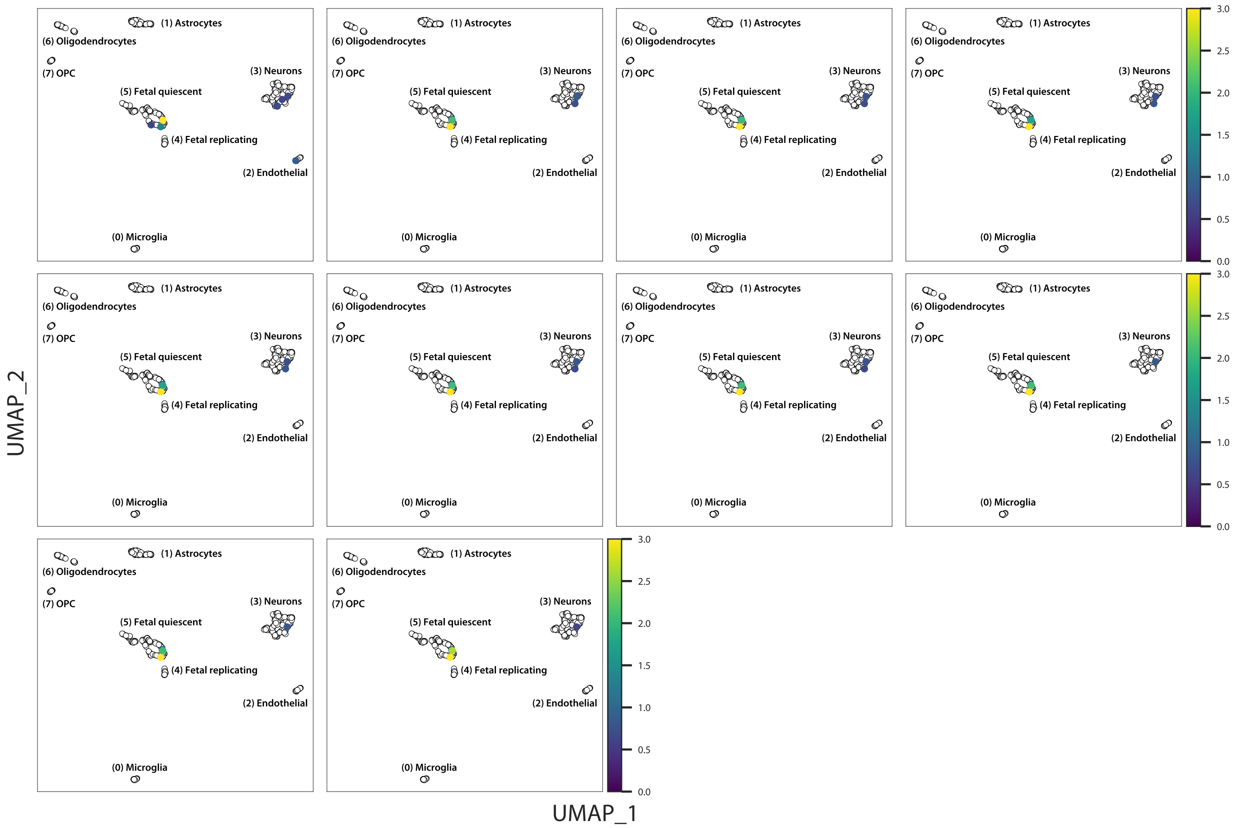

Supplementary Figure 25: Difference between each head's values and the mean, for all 16 attention heads of the Darmanis CellVGAE model corresponding to the mean (μ) layer.

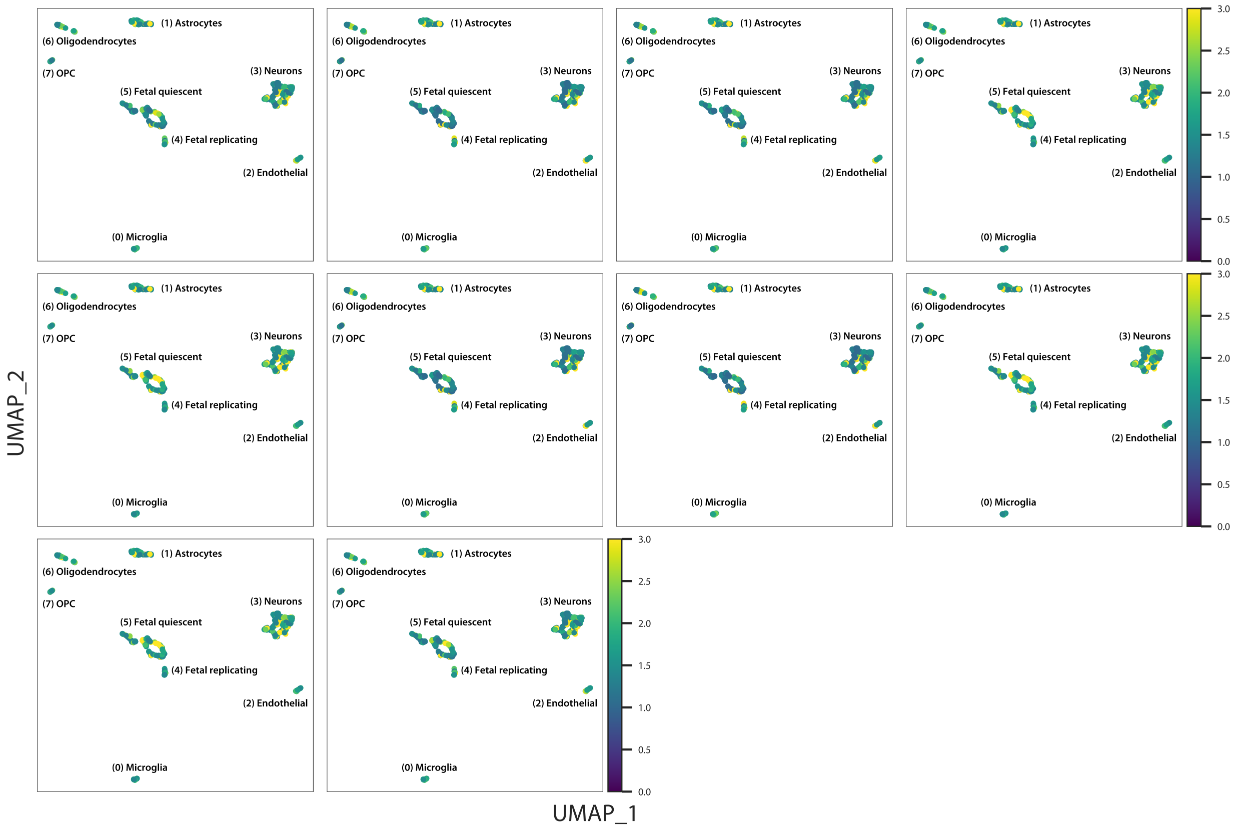

Supplementary Figure 26: All 16 attention heads of the Darmanis CellVGAE model corresponding to the std (σ) layer.

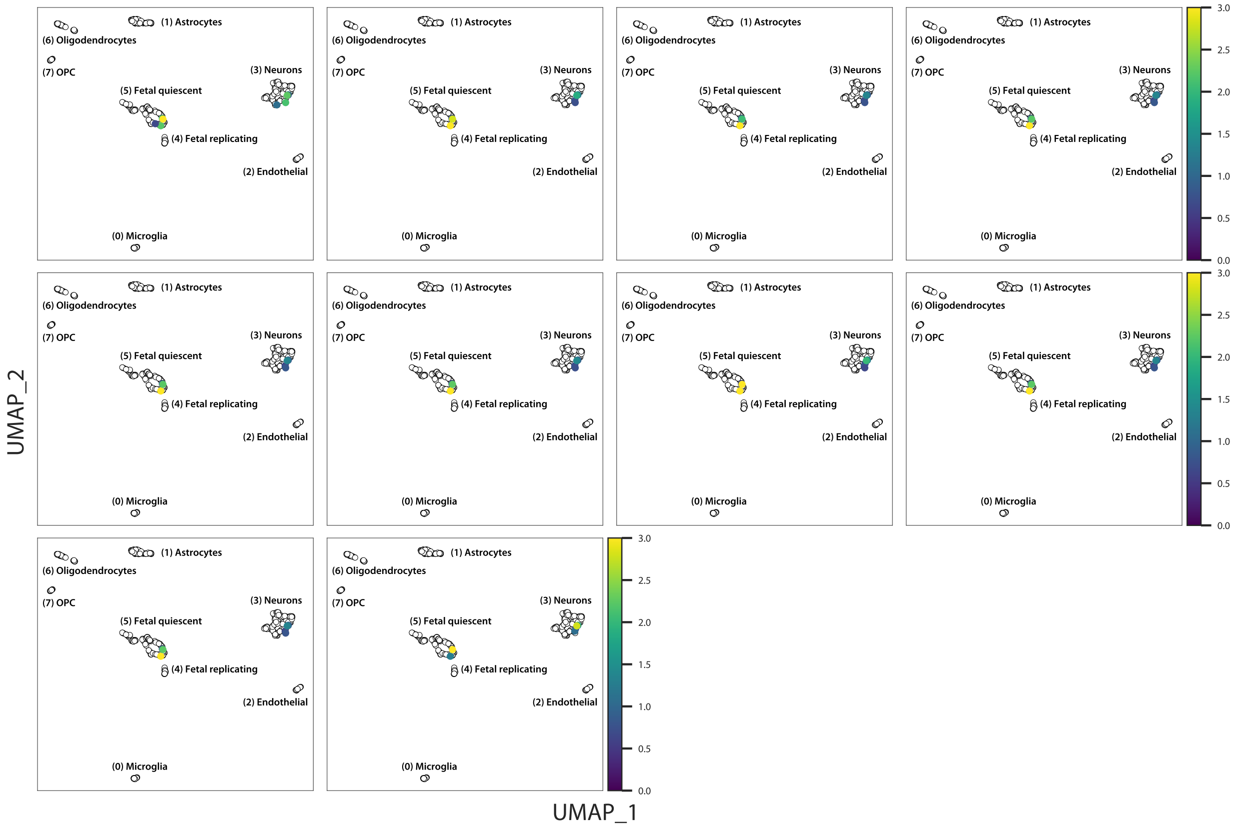

Supplementary Figure 27: Difference between each head's values and the mean, for all 16 attention heads of the Darmanis CellVGAE model corresponding to the std (σ) layer.

Supplementary Information References

[37] Lun, A. T. L., McCarthy, D. J. & Marioni, J. C. A step-by-step workflow for low-level analysis of single-cell rna-seq data with bioconductor. *F1000Res.* **5**, 2122 (2016).

[38] Hubert, L. & Arabie, P. Comparing partitions. *Journal of Classification* **2**, 193–218 (1985). URL https://doi.org/10.1007/BF01908075.

[39] Darmanis, S. *et al.* A survey of human brain transcriptome diversity at the single cell level. *Proceedings of the National Academy of Sciences* **112**, 7285–7290 (2015). URL https://www.pnas.org/content/112/23/7285. https://www.pnas.org/content/112/23/7285.full.pdf.

[40] Wang, Y. J. *et al.* Single-cell transcriptomics of the human endocrine pancreas. *Diabetes* **65**, 3028–3038 (2016). URL https://pubmed.ncbi.nlm.nih.gov/27364731. 27364731[pmid].

[41] Baron, M. *et al.* A single-cell transcriptomic map of the human and mouse pancreas reveals inter- and intra-cell population structure. *Cell Systems* **3**, 346–360.e4 (2016). URL https://doi.org/10.1016/j.cels.2016.08.011.

[42] Loh, K. M. *et al.* Mapping the pairwise choices leading from pluripotency to human bone, heart, and other mesoderm cell types. *Cell* **166**, 451–467 (2016). URL https://doi.org/10.1016/j.cell.2016.06.011.

[43] Segerstolpe, Å. *et al.* Single-cell transcriptome profiling of human pancreatic islets in health and type 2 diabetes. *Cell metabolism* **24**, 593–607 (2016). URL https://pubmed.ncbi.nlm.nih.gov/27667667. 27667667[pmid].

[44] Muraro, M. *et al.* A single-cell transcriptome atlas of the human pancreas. *Cell Systems* **3**, 385–394.e3 (2016). URL https://doi.org/10.1016/j.cels.2016.09.002.

[45] Shekhar, K. *et al.* Comprehensive classification of retinal bipolar neurons by single-cell transcriptomics. *Cell* **166**, 1308–1323.e30 (2016). URL https://doi.org/10.1016/j.cell.2016.07.054.

[46] Paszke, A. *et al.* Pytorch: An imperative style, high-performance deep learning library. In Wallach, H. *et al.* (eds.) *Advances in Neural Information Processing Systems 32*, 8024–8035 (Curran Associates, Inc., 2019). URL http://papers.neurips.cc/paper/9015-pytorch-an-imperative-style-high-performance-deep-learning-library.pdf.

[47] Fey, M. & Lenssen, J. E. Fast graph representation learning with PyTorch Geometric. In *ICLR Workshop on Representation Learning on Graphs and Manifolds* (2019).

[48] Hunter, J. D. Matplotlib: A 2d graphics environment. *Computing in Science & Engineering* **9**, 90–95 (2007).

[49] Waskom, M. & the seaborn development team. mwaskom/seaborn (2020). URL `https://doi.org/10.5281/zenodo.592845`.

[50] Inc., P. T. Collaborative data science (2015). URL `https://plot.ly`.

[51] R Core Team. *R: A Language and Environment for Statistical Computing*. R Foundation for Statistical Computing, Vienna, Austria (2013). URL `http://www.R-project.org/`.
